# Long-term engrafting multilineage hematopoietic cells differentiated from human induced pluripotent stem cells

**DOI:** 10.1038/s41587-024-02360-7

**Published:** 2024-09-02

**Authors:** Elizabeth S. Ng, Gulcan Sarila, Jacky Y. Li, Hasindu S. Edirisinghe, Ritika Saxena, Shicheng Sun, Freya F. Bruveris, Tanya Labonne, Nerida Sleebs, Alexander Maytum, Raymond Y. Yow, Chantelle Inguanti, Ali Motazedian, Vincenzo Calvanese, Sandra Capellera-Garcia, Feiyang Ma, Hieu T. Nim, Mirana Ramialison, Constanze Bonifer, Hanna K. A. Mikkola, Edouard G. Stanley, Andrew G. Elefanty

**Affiliations:** 1 Murdoch Children’s Research Institute, The Royal Children’s Hospital, Parkville, Victoria Australia; 2 Department of Paediatrics, Faculty of Medicine, Dentistry and Health Sciences, University of Melbourne, Parkville, Victoria Australia; 3 The Novo Nordisk Foundation Center for Stem Cell Medicine (reNEW), Murdoch Children’s Research Institute, Parkville, Victoria Australia; 4 Institute for Cancer and Genomic Sciences, College of Medical and Dental Sciences, University of Birmingham, Birmingham, UK; 5 Cancer Immunology Program, Peter MacCallum Cancer Centre, Melbourne, Victoria Australia; 6 Sir Peter MacCallum Department of Oncology, The University of Melbourne, Parkville, Victoria Australia; 7 Department of Molecular, Cell and Developmental Biology, University of California, Los Angeles, Los Angeles, CA USA; 8 Eli and Edythe Broad Center for Regenerative Medicine and Stem Cell Research, University of California, Los Angeles, Los Angeles, CA USA; 9 Laboratory for Molecular Cell Biology, University College London, London, UK; 10 Australian Regenerative Medicine Institute, Monash University, Clayton, Victoria Australia; 11 Present Address: Changping Laboratory, Beijing, China

**Keywords:** Induced pluripotent stem cells, Cell biology, Haematopoietic stem cells

## Abstract

Hematopoietic stem cells (HSCs) derived from human induced pluripotent stem cells (iPS cells) have important biomedical applications. We identified differentiation conditions that generate HSCs defined by robust long-term multilineage engraftment in immune-deficient NOD,B6.*Prkdc*^*scid*^ *Il2rg*^*tm1Wjl/SzJ*^ *Kit*^*W41/W41*^ mice. We guided differentiating iPS cells, as embryoid bodies in a defined culture medium supplemented with retinyl acetate, through *HOXA*-patterned mesoderm to hemogenic endothelium specified by bone morphogenetic protein 4 and vascular endothelial growth factor (VEGF). Removal of VEGF facilitated an efficient endothelial-to-hematopoietic transition, evidenced by release into the culture medium of CD34^+^ blood cells, which were cryopreserved. Intravenous transplantation of two million thawed CD34^+^ cells differentiated from four independent iPS cell lines produced multilineage bone marrow engraftment in 25–50% of immune-deficient recipient mice. These functionally defined, multipotent CD34^+^ hematopoietic cells, designated iPS cell-derived HSCs (iHSCs), produced levels of engraftment similar to those achieved following umbilical cord blood transplantation. Our study provides a step toward the goal of generating HSCs for clinical translation.

## Abstract (web summary)

Human hematopoietic cells derived from pluripotent cells repopulate multiple blood lineages in mice.

## Main

The differentiation of human pluripotent stem cells (PS cells) into repopulating hematopoietic stem cells (HSCs) could provide novel therapeutic options for a range of hematopoietic disorders. For example, HSCs derived from patient induced PS cells (iPS cells)^1^ could circumvent the donor–host mismatch that leads to graft-versus-host-disease, a major source of morbidity and mortality in recipients of imperfectly matched allogenic transplants^2^. HSCs derived from genome-edited iPS cells could treat patients by correcting genetic causes of blood diseases, such as bone marrow failure syndromes^3^. Modeling of hematopoietic development or diseases using gene-edited iPS cell-derived cells^4–8^ could accurately recapitulate aberrant hematopoiesis, thereby facilitating the development of more effective therapies.

The earliest human blood cells develop extraembryonically in distinct waves from the yolk sac (YS)^9,10^. Intraembryonic hematopoietic cells, whose descendants form the adult blood system, develop separately in the aorta–gonad–mesonephros (AGM) region, where they bud from the aortic wall between days 27 and 40 of gestation (Carnegie stages (CS) 13–17)^10,11^. Following their emergence from the aorta, these preHSCs mature and colonize the fetal liver and, in the process, acquire robust repopulating capacity^10^. The functional HSC pool then expands in the fetal liver before seeding the bone marrow^10^. Although the AGM of day 32–41 (CS14–17) human embryos generates predominantly preHSCs, it also produces infrequent repopulating HSCs (~1 per embryo)^12^.

The generation of repopulating HSCs from PS cells has proved challenging, partly because of difficulties in distinguishing cells representing AGM-type hematopoiesis from those similar to the YS that cannot engraft. However, it was found that *HOXA* gene expression could be distinguished between YS-derived *HOXA*-negative and AGM-derived *HOXA*-positive cells, providing a critical marker for guiding directed differentiation of iPS cells to repopulating HSCs^13,14^. Subsequently, we and others found that culturing the mesoderm with the Wnt agonist CHIR99201 (refs. ^15,16^) and/or the anaplastic lymphoma kinase (ALK) inhibitor SB431542 (ref. ^17^) patterned cells to a *HOXA*-positive state, mimicking an AGM-like differentiation trajectory^18^. The gene expression pattern of iPS cell-derived cells following this trajectory resembled that of cells in the AGM of day 32 embryos (CS14), when the first repopulating human HSCs arise^13,18^. However, it was not known whether this similarity in gene expression would also translate into a functional similarity.

Here, we established an iPS cell differentiation protocol that generated CD34^+^ HSCs (denoted iHSCs) capable of multilineage engraftment (MLE). Key elements of the protocol included a defined medium and cryopreservation of CD34^+^ cells for compatibility with future clinical applications. Our experiments revealed that the endowment of CD34^+^ hematopoietic cells with MLE capacity in immune-deficient mice depended on the timed provision of Wnt agonists, retinoic acid precursors and vascular endothelial growth factor (VEGF), reflecting the roles of these molecules in the specification of the hematopoietic system^19–21^. These studies lay the groundwork for further dissection of HSC formation from iPS cells and eventual clinical translation.

## Results

### Differentiation of iPS cells to CD34-expressing hematopoietic cells

**Fig. 1:**
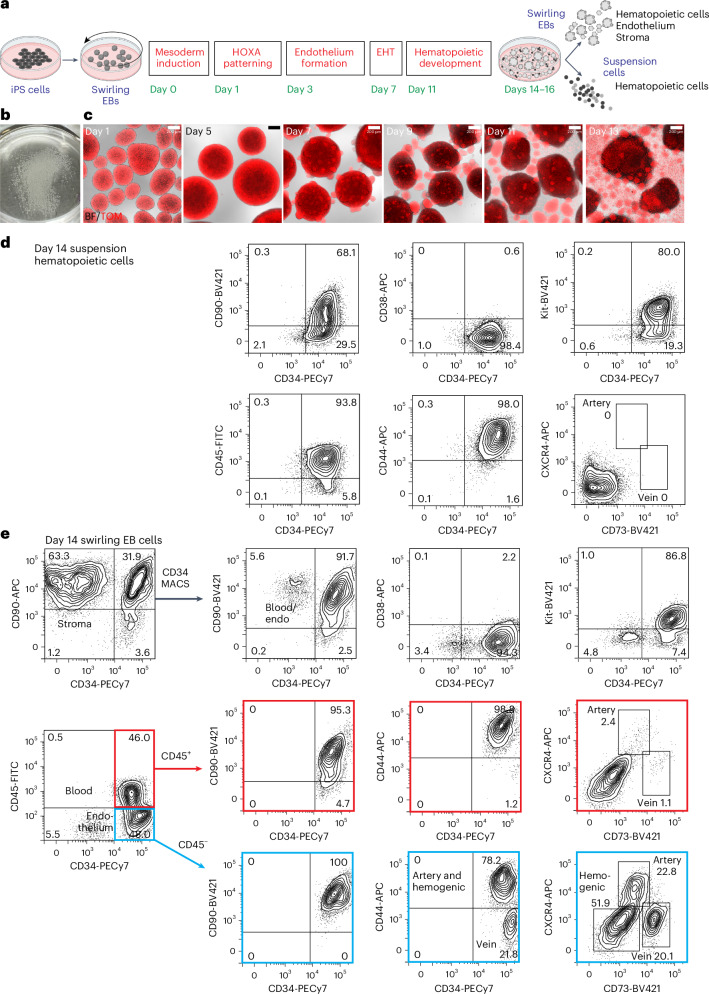
In vitro hematopoietic differentiation of iPS cells. **a**, Swirling EB differentiation protocol indicating differentiation stages transitioning from undifferentiated iPS cells to hematopoietic, endothelial and stromal cells. Growth factors for each stage are shown in Extended Data Fig. 1a and the Methods. EHT, endothelial-to-hematopoietic transition. Partially created using BioRender.com. **b**, A 60-mm dish on day 7 showing hundreds of swirling EBs. **c**, Overlaid bright-field (BF) and tandem TOMATO (TOM) fluorescence images of developing swirling EB cultures. Scale bar, 200 µm. **d**, Flow cytometry of day 14 suspension hematopoietic cells showing the expression of surface CD45, CD34, Kit, CD44 and CD90. **e**, Dissociated day 14 swirling EB cells were typically enriched to >90% CD34^+^ endothelium and blood using MACS. These cells comprised CD45^+^ blood cells (profiles with red borders) and CD45^−^ endothelium (profiles with blue borders). The endothelium was categorized as arterial, venous or hemogenic on the basis of the expression of CD34, CD44, CXCR4 and CD73 (ref. ^15^). The flow cytometry results in **d**,**e** are from one representative experiment of more than 20 experiments performed.

**Extended Data Fig. 1:**
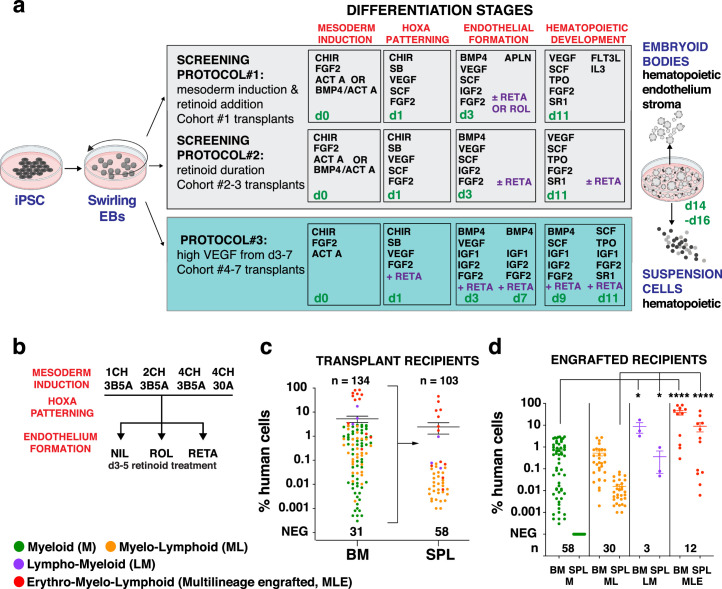
iPSC differentiation protocols and transplantation results for cohort 1. (**a**) Schematic outline of the growth factors used for iPSC differentiation in screening protocols 1 and 2 and in protocol 3. Cohorts of mice transplanted with each protocol are indicated. Concentrations of growth factors used are provided in Methods. Partially created using BioRender.com. (**b**) Combinations of mesoderm induction factors provided during the first day of differentiation in screening protocol 1, and retinoids during endothelium formation from day 3 to day 5, generated 12 differentiation conditions transplanted into cohort 1 mice. See also Fig. 2a. Concentration of CHIR (CH) is in µM, and concentrations of BMP4 (B) and ACTIVIN A (A) are in ng/ml. (**c**) Bone marrow (BM) and spleen (SPL) engraftment in 134 cohort 1 transplant recipients. Time of analysis is shown in Supplementary Tables 2 and 3. Each circle represents one animal, color coded to indicate myeloid (M), myelo-lymphoid (ML), lympho-myeloid (LM) and erythro-myelo-lymphoid multilineage (MLE) patterns of engraftment. Total number of mice is shown, as is number of unengrafted (NEG) mice. Error bars, mean ± s.e.m. (**d**) Engrafted recipients categorized by engraftment phenotype demonstrate higher levels of human cells in the BM and SPL of lympho-myeloid and multilineage engrafted animals. Number of mice with each phenotype is shown. BM: M vs LM, * *P* = 0.0175; M vs MLE, **** *P* < 0.0001, one-way ANOVA (Kruskal-Wallis) test with Dunn’s multiple comparisons test. SPL: ML SPL vs LM SPL, * *P* = 0.0447; ML SPL vs MLE SPL, **** *P* < 0.0001, one-way ANOVA (Kruskal-Wallis) test with Dunn’s multiple comparisons test. Error bars, mean ± s.e.m. Source data

For all differentiation protocols, iPS cells were dissociated and seeded into dishes that were incubated on a rotating platform, allowing the formation of swirling embryoid bodies (EBs) that differentiated to hematopoietic cells^13,22^ (Fig. 1a,b and Supplementary Results 1; see Extended Data Fig. 1a for an overview of differentiation protocols and Methods for details of growth factor combinations). Mesoderm was induced for 24 h, patterned for 2 days to induce the expression of *HOXA* genes^18^ and differentiated to hemogenic endothelium from days 3 to 7. Cells undergoing an endothelial-to-hematopoietic transition protruded from the surface of the EBs, reminiscent of intra-arterial hematopoietic clusters of blood cells emerging from the embryonic aorta^10,11^ (Fig. 1c). These cellular accumulations broke away from the EBs, shedding blood cells into the medium from day 11 (Fig. 1c). Cultures on day 14 comprised a dominant blood cell suspension with most cells expressing CD34, CD90, CD44 and Kit (Fig. 1d and Supplementary Fig. 1). The EB-derived fraction consisted of the stroma, the endothelium and hematopoietic cells that had not yet shed into the medium (Fig. 1e and Supplementary Fig. 1). A small proportion of the hematopoietic cells expressed CXCR4 or CD73, reflecting their recent emergence from an endothelial precursor (Fig. 1e). From days 14 to 16, the suspension hematopoietic cells were harvested and cryopreserved (Fig. 1d). In some experiments, CD34^+^ cells enriched from EBs by magnetic-activated cell sorting (MACS) (Fig. 1e) were also cryopreserved.

### MLE cells require retinoids during iPS cell differentiation

**Fig. 2:**
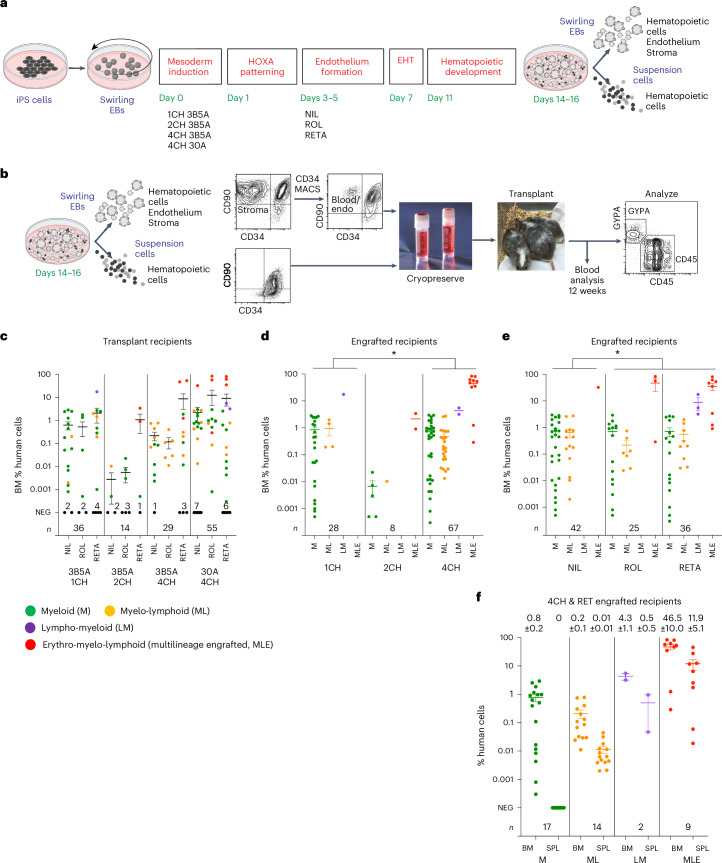
MLE depends on CHIR and retinoids during iPS cell differentiation. **a**, Swirling EB differentiation protocol (screening protocol 1; Extended Data Fig. 1a) indicating mesoderm induction factors provided during the first day of differentiation and retinoids during endothelium formation from days 3 to 5 to generate the 12 differentiation conditions transplanted into mice in cohort 1. Numbers indicate the concentration of CHIR (CH) in µM and concentrations of BMP4 (B) and Activin A (A) in ng ml^−1^. **b**, Transplantation workflow showing the cryopreservation of CD34^+^ hematopoietic cells from the cell suspension along with MACS-isolated CD34^+^ cells from the EB. MACS-enriched EB cells were not collected for all experiments. Cryopreserved cells were thawed and transplanted immediately into NBSGW immune-deficient mice by tail-vein injection. Peripheral blood was analyzed at 12 weeks to screen for engraftment and hematopoietic tissues were analyzed for human cells at time points up to 24 weeks (Supplementary Tables 2 and 3). **c**, Scatter dot plot correlating the percentage of bone marrow (BM) human cells with differentiation conditions in cohort 1. Error bars, mean ± s.e.m. The number of mice receiving cells subjected to each mesoderm induction (*n*) is shown. The number of unengrafted (NEG) mice is indicated for each condition. **d**, Scatter dot plot correlating the concentration of CHIR during mesoderm induction with the phenotype of engrafted human cells in the BM (colored circles). The number of mice displaying an MLE phenotype differed between those receiving cells treated with 4CH and 1CH. *P = 0.03, determined by a two-sided Fisher’s exact test. Error bars, mean ± s.e.m. Data from the 4CH 3B5A and 4CH 30A mesoderm inductions were pooled. **e**, Scatter dot plot correlating the inclusion of retinoid (ROL or RETA) during iPS cell differentiation with the phenotype of engrafted human cells in the BM (colored circles). The number of mice displaying an MLE phenotype differed between those receiving cells treated with or without retinoid. ROL or RETA versus NIL (no retinoid). *P = 0.03, determined by a two-sided Fisher’s exact test. Error bars, mean ± s.e.m. Data from the 4CH 3B5A and 4CH 30A mesoderm inductions were pooled. **f**, Phenotypes in 42/51 mice transplanted with cells treated with the combination of 4 µM CHIR and retinoid (RET) that showed engraftment. In total, 9/51 (17.6%) transplanted mice showed MLE. Error bars, mean ± s.e.m. Source data

We screened combinations of CHIR, Activin A, bone morphogenetic protein 4 (BMP4) and a retinoid during the mesoderm induction and patterning stages (screening protocol 1; Methods and Extended Data Fig. 1a,b) to determine whether any supported the generation of engraftable human hematopoietic cells. CD34^+^ hematopoietic cells were generated from an iPS cell line constitutively expressing a tandem TOMATO fluorescent protein (RM TOM) (Fig. 1c)^23^ and cryopreserved before thawing and injection into the tail vein of NOD,B6.*Prkdc*^*scid*^ *Il2rg*^*tm1Wjl/SzJ*^ *Kit*^*W41/W41*^ (NBSGW) mice^24^, mimicking the workflow of clinical HSC transplantation (Fig. 2a,b). In this series of experiments, groups of mice (totaling 134, denoted cohort 1) were injected with cells differentiated under one of 12 mesoderm induction and patterning protocols in screening protocol 1 (Supplementary Results 2, Fig. 2a–f, Extended Data Fig. 1b–d and Supplementary Tables 1–3 and 13). Some mice (12/134) were engrafted by stem cells displaying multilineage differentiation resulting in erythroid, myeloid and lymphoid reconstitution (denoted MLE). We found that most mice in which MLE occurred received cells in which the mesoderm was induced with 4 µM CHIR on day 0 and a pulse of a retinoic acid precursor (retinol (ROL) or retinyl acetate (RETA)) was included from days 3 to 5 of differentiation (Fig. 2a–e). Indeed, 17.6% (9/51) of mice transplanted with cells treated with the combination of 4 µM CHIR and retinoid showed MLE (Fig. 2f). There were over 80% human cells occupying the bone marrow in some of these MLE cohort 1 recipients (average: 46.5% ± 10.0% human cells in bone marrow and 11.9% ± 5.1% in spleen) (Fig. 2f and Supplementary Tables 1 and 13), highlighting the capacity for differentiation of the engrafting cells. All MLE mice in cohort 1 and subsequent transplant cohorts were engrafted with ≥0.1% human cells (Supplementary Tables 13 and 19). Hereafter, we refer to these functionally defined iPS cell-derived multipotent hematopoietic cells with the capacity to engraft multiple lineages over a long term as ‘iHSCs’.

### Transcriptional similarity of in vitro differentiated iPS cells and human AGM

**Fig. 3:**
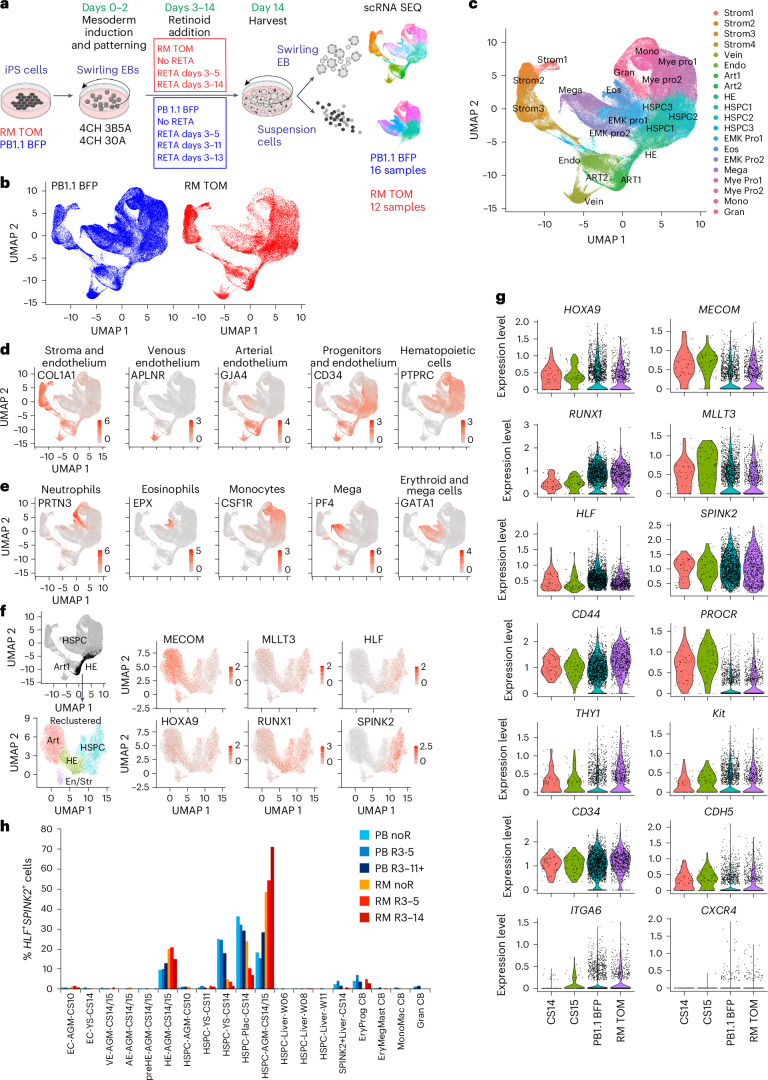
Transcriptional profiling of in vitro differentiated iPS cells. **a**, Swirling EB differentiation protocol showing the mesoderm induction and retinoid combinations used to differentiate RM TOM and PB1.1 BFP iPS cells. Each cell line was subjected to two mesoderm induction conditions, with 4 µM CHIR, 3 ng ml^−1^ BMP4 and 5 ng ml^−1^ Activin A (4CH 3BA5) or 4 µM CHIR and 30 ng ml^−1^ Activin A (4CH 30A), and three or four RETA exposure patterns. Samples were isolated from swirling EB and suspension hematopoietic cell fractions on day 14 of differentiation, leading to 28 samples subjected to scRNA seq. Partially created using BioRender.com. **b**,**c**, UMAP of integrated samples for individual lines (**b**) and following pooling of samples (**c**), showing the annotation of cell clusters allocated on the basis of cluster-specific gene expression (Supplementary Table 4). **d**,**e**, Feature plots depicting selected genes identifying tissue types (**d**) and hematopoietic cell lineages (**e**) in integrated samples. **f**, Feature plots depicting the expression of six human HSC signature genes^13^ in arterial (Art1), endothelial or stromal (En/Str), hemogenic (HE) and *HLF*^+^*SPINK2*^+^ cells from HSPC clusters 1–3 in integrated samples. The cell numbers and composition of clusters are provided in Supplementary Table 5. **g**, Violin plots showing the expression of selected stem cell genes in *HLF*^+^*SPINK2*^+^ cells from the HSPC cluster in CS14 and 15 embryos and from *HLF*^+^*SPINK2*^+^ cells from HSPC cluster 1 (**c**) in PB1.1 BFP and RM TOM cells. Cell numbers: CS14, 51; CS15, 70; PB1.1 BFP, 2,983; RM TOM, 1,112 (Supplementary Table 5). **h**, Comparison of the expression profiles of *HLF*^+^*SPINK2*^+^ cells from HSPC clusters from PB1.1 BFP and RM TOM cells to reference data from human embryonic-derived and CB-derived endothelial and hematopoietic cell populations, using the ACTINN machine learning algorithm to determine the percentage of iPS cell-derived hematopoietic cells displaying the greatest similarity to each reference dataset. Data stratified by retinoid treatment are shown for each cell line. The bar height represents the percentage of *HLF*^+^*SPINK2*^+^ putative iHSCs that map most closely to each reference sample. Cell numbers mapping to each reference sample are shown in Supplementary Table 10. EC, endothelial cell; VE, venous endothelium; AE, arterial endothelium; preHE, prehemogenic endothelium (representing aortic endothelium); HE, hemogenic endothelium; W, week; Plac, placenta; Ery, erythroid; Prog, progenitor; Meg, megakaryocyte; Mast, mast cell; Mono, monocyte; Mac, macrophage; Gran, granulocyte.

To search for transcriptional signatures accounting for functional differences between cells differentiated with and without retinoid and to allow comparisons to published datasets of human AGM, we performed single-cell RNA sequencing (scRNA seq) of differentiated iPS cells^13,25^. Two iPS cell lines were profiled, the RM TOM line described above and an independent line in which the mTagBFP2 fluorescent protein^23^ was expressed from the *GAPDH* locus of PB1.1 iPS cells^26^ (denoted PB1.1 BFP). Mesoderm was induced with 4 µM CHIR and 3 ng ml^−1^ BMP4 and 5 ng ml^−1^ Activin A (4CH 3B5A) or 30 ng ml^−1^ Activin A (4CH 30A), because our transplant results in cohort 1 identified that these conditions supported the generation of MLE mice (Fig. 2c). Because embryo data indicate that AGM-derived HSCs develop in a retinoid-replete milieu^20^, cultures were treated with RETA from days 3 to 5 (as was the case in cohort 1) or for a more prolonged period where RETA was added every 2 days from days 3 to 11, 13 or 14) (Fig. 3a). Control cultures were differentiated without added RETA. After 14 days, blood cells in suspension and disaggregated EBs were subjected to scRNA seq using the 10X Genomics platform. In total, 252,607 cells, comprising 12 RM TOM and 16 PB1.1 BFP samples, were analyzed. Uniform manifold approximation projection (UMAP) plots of integrated samples from both cell lines, followed by cluster analysis, allowed the allocation of cells to stromal, endothelial, hemogenic and hematopoietic lineages (Fig. 3b–e and Supplementary Table 4). The diverse lineages identified in scRNA seq indicate the considerable heterogeneity already present in the cultures, which was not obvious from the flow cytometric phenotype.

**Extended Data Fig. 2:**
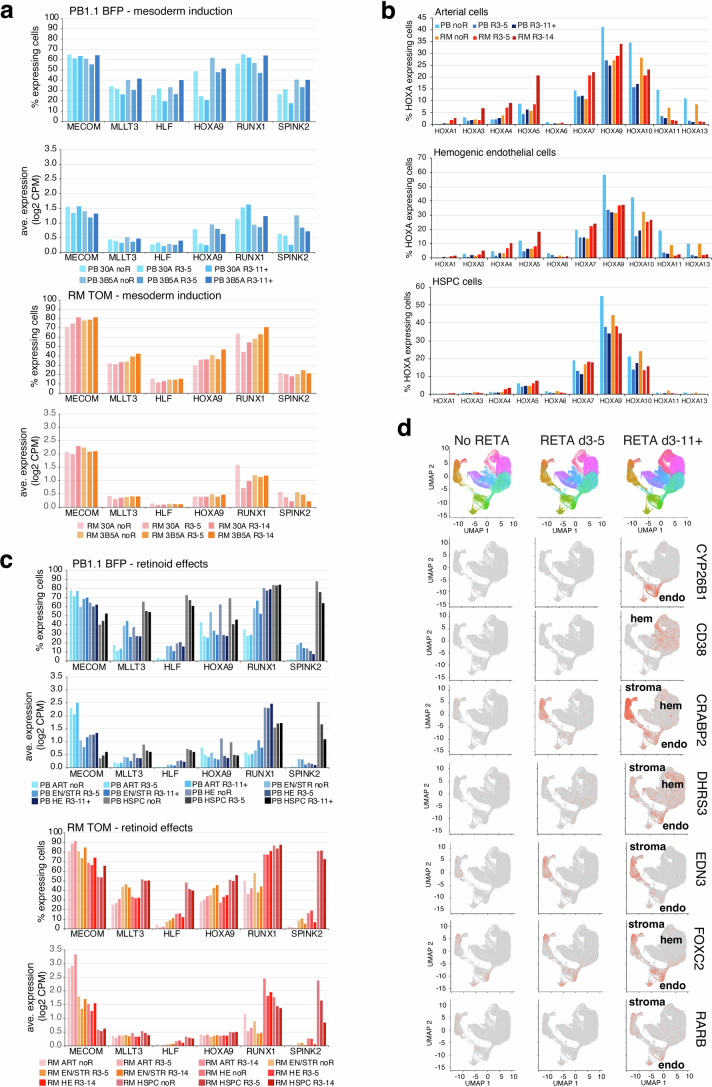
Effects of retinoids and mesoderm induction protocols on expression of stem cell genes during the endothelial to hematopoietic transition in vitro and expression of retinoid dependent genes in iPSC-differentiated cells. (**a**) Bar graphs displaying the expression of HSC signature genes in cells encompassing the endothelial to hematopoietic transition (see Fig. 3f), differentiated from RM TOM and PB1.1 BFP iPSC lines, under three retinyl acetate (RETA) conditions and two mesoderm induction protocols. no R, no RETA; R3–5, RETA day 3−5; R3–11 + , RETA day 3-11, 3−13, or 3−14. Both the percentage of positive cells and the average expression of each gene is shown. Cell numbers and composition are provided in Supplementary Table 5. (**b**) Bar graphs displaying the percentage of cells expressing *HOXA* genes in arterial, hemogenic endothelium and hematopoietic stem and progenitor (HSPC) cells differentiated from RM TOM and PB1.1 BFP iPSC lines under three RETA conditions. Cell numbers and composition are provided in Supplementary Table 5. (**c**) Bar graphs displaying the expression of HSC signature genes in cells differentiated from RM TOM and PB1.1 BFP iPSC lines, shown for each cluster (see Fig. 3f) under three RETA conditions. no R, no RETA; R3–5, RETA day 3-5; R3–11 + , RETA day 3-11, 3−13, or 3−14. Both the percentage of positive cells and the average expression of each gene is shown. Cell numbers and composition are provided in Supplementary Table 5. (**d**) Feature plots showing expression of selected retinoid responsive genes in day 14 differentiated human iPSCs correlated with RETA exposure. Integrated data from 4CH 3B5A and 4CH 30 A mesoderm inductions was pooled. Endothelial (endo), hematopoietic (hem) and stromal (stroma) populations indicated.

Reclustering cells within arterial, hemogenic and *HLF*^+^*SPINK2*^+^ cells within the HSC clusters confirmed that the HSC signature genes (*RUNX1*, *MECOM*, *MLLT3*, *HLF*, *HOXA9* and *SPINK2*) recently identified in the human AGM^13^ (Supplementary Fig. 2a,b) were also expressed in iPS cell-derived cells (Fig. 3f and Supplementary Table 5). The percentage of cells expressing HSC signature genes and the level of expression of these genes were very similar in the RM TOM and PB1.1 BFP cell lines under both mesoderm induction conditions (4CH 3B5A and 4CH 30A) (Extended Data Fig. 2a). We also confirmed the expected pattern of *HOXA* gene expression in response to the SB and CHIR patterning in both cell lines (Extended Data Fig. 2b).

The addition of retinoids minimally impacted stem cell gene expression (Extended Data Fig. 2c) but notably influenced genes associated with retinoic acid metabolism such as *CYP26B1*, *DHRS3*, *CRABP2*, *RARB* and *RARG*, modulators of Wnt and fibroblast growth factor (FGF) signaling such as *SHISA3*, *DKK1*, *RSPO1* and *WNT4*, as well as genes associated with vascular and hematopoietic development such as *FOXC2* and *CD38* (Supplementary Results 3, Supplementary Fig. 3 and Supplementary Tables 6–9). Many retinoid-responsive genes were only induced if the retinoids were included until at least day 11 of differentiation (Extended Data Fig. 2d).

**Extended Data Fig. 3:**
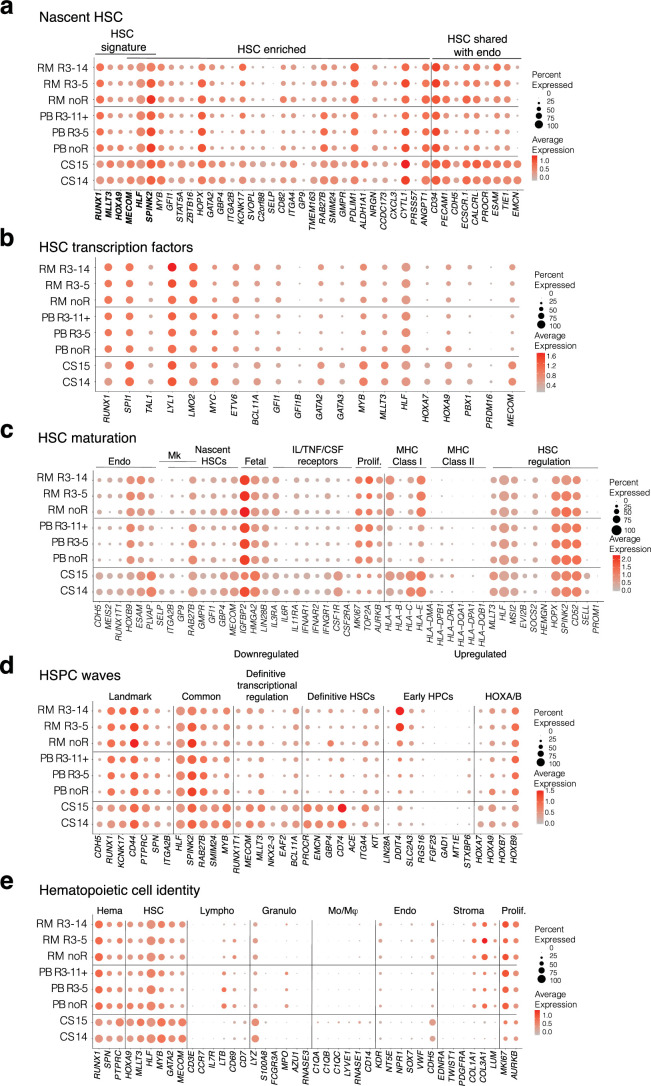
Comparison of the transcriptomes of iPSC-derived cells from the HSPC clusters that co-expressed *HLF* and *SPINK2*, with those of *HLF*^+^*SPINK2*^+^ cells from CS14 and CS15 embryos (Part 1). (**a**) Nascent HSC. (**b**) HSC transcription factors. (**c**) HSC maturation. (**d**) HSPC waves. (**e**) Hematopoietic cell identity. Cell numbers: CS14, 51; CS15, 70; PB noR, 489; PB R3-5, 880; PB R3-11 + , 1614; RM noR, 424; RM R3-5, 400; RM R3-14, 288. See also Supplementary Table 5. Abbreviations: PB, PB1.1 BFP; RM, RM TOM. The scorecards developed by the Mikkola laboratory were used as templates^13^.

**Extended Data Fig. 4:**
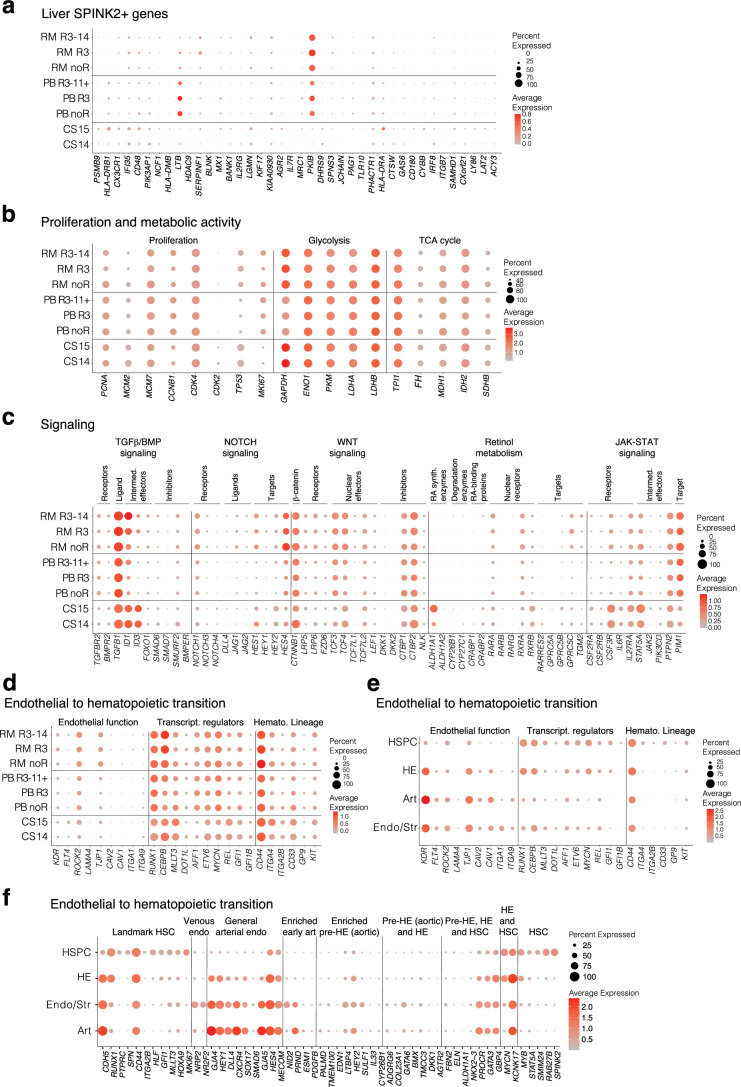
Comparison of the transcriptomes of iPSC-derived cells from the HSPC clusters that co-expressed *HLF* and *SPINK2*, with those of *HLF*^+^*SPINK2*^+^ cells from CS14 and CS15 embryos (Part 2). (**a**) Liver *SPINK2*^+^ genes. (**b**) Proliferation and metabolic activity. (**c**) Signaling. (**d**–**f**) Endothelial to hematopoietic transition. Samples for panels (e) and (f) are the clusters shown in Fig. 3f. Cell numbers for panels (a)–(d): CS14, 51; CS15, 70; PB noR, 489; PB R3-5, 880; PB R3-11 + , 1614; RM noR, 424; RM R3-5, 400; RM R3-14, 288. See also Supplementary Table 5. Abbreviations: PB, PB1.1 BFP; RM, RM TOM. The scorecards developed by the Mikkola laboratory were used as templates^13^.

We compared the transcriptional profiles of the iPS cell-derived *HLF*^+^*SPINK2*^+^ cells to similar *HLF*^+^*SPINK2*^+^ stem cell-like populations from human embryos at CS14 and CS15, examining the expression of a selected range of relevant genes (Fig. 3g). For a more extensive comparison between in vitro and human embryo-derived samples, we made use of the suite of scorecards developed in profiling studies of hematopoietic development in human embryos^13^ (Supplementary Results 4 and Extended Data Figs. 3 and 4). These studies benchmarked our iPS cell *HLF*^+^*SPINK2*^+^ cells against *HLF*^+^*SPINK2*^+^ CS14 and CS15 human embryo cells, demonstrating a high level of concordance between the transcriptional profiles across the nine scorecards of genes examined.

Lastly, we used a machine learning algorithm, ACTINN^27^, to compare the expression profiles of day 14 differentiated iPS cells to a human reference dataset comprising hematovascular cells from gestational day 22 to 24 (CS10–11) embryo and YS, day 29–36 (CS14–15) AGM, YS, embryonic liver and placenta and week 6, 8, 11 and 15 embryonic and fetal liver hematopoietic stem and progenitor cells (HSPCs) and cord blood (CB) stem and progenitor cells^13^. This analysis confirmed that *HLF*^+^*SPINK2*^+^ cells were most closely related to cells categorized as HSPCs in CS14–15 AGM, placenta and YS (Fig. 3h and Supplementary Table 10). Dissecting the allocation of cells to these categories from the two cell lines and the different durations of retinoid treatment revealed that cells derived from the RM TOM line mapped predominantly to the CS14–15 AGM HSPCs, whilst the PB1.1 BFP cells were more similar to CS14 YS and placental HSPCs. We can only speculate whether the greater overall proportion of RM TOM cells mapping to the CS14–15 AGM HSC sample was of functional importance. For both lines, the longer duration of retinoid increased the proportion of CS14–15 AGM HSPCs and decreased the CS14 YS and placental HSPCs (Fig. 3h).

### MLE cells are generated from cultures treated with retinoids throughout differentiation

**Fig. 4:**
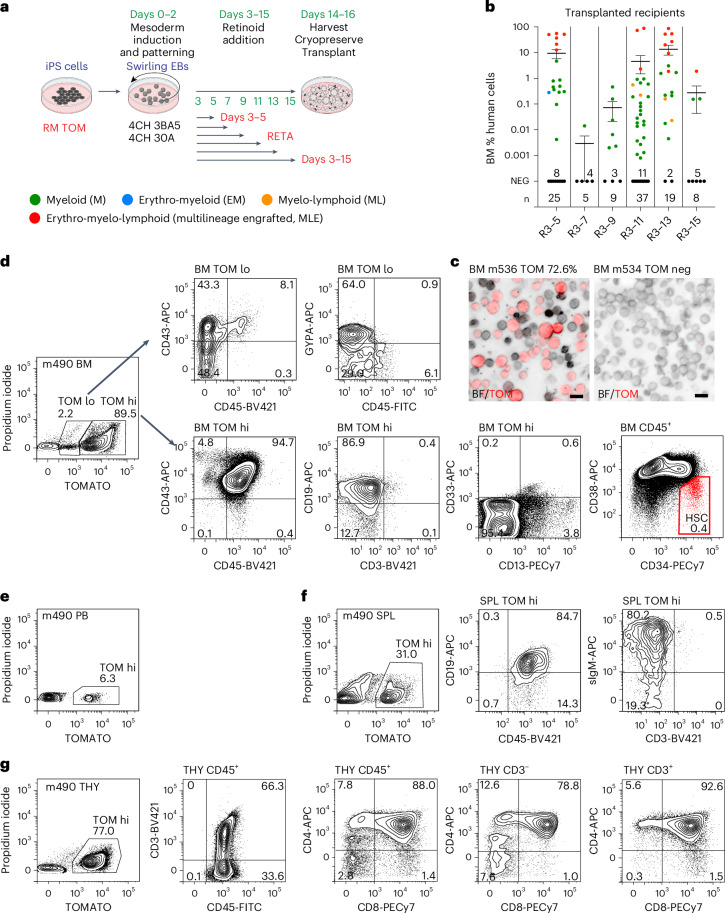
Hematopoietic cells exposed to retinoid throughout differentiation possess MLE potential. **a**, Swirling EB differentiation protocol (screening protocol 2; Extended Data Fig. 1a) showing the mesoderm induction and retinoid combinations used to differentiate RM TOM iPS cells for cohort 2 transplants. Cells were subjected to two mesoderm induction conditions and six retinoid exposure patterns before harvesting and cryopreservation on days 14–16. Partially created using BioRender.com. **b**, Scatter dot plot correlating human cells in the BM with the interval of retinoid (R) treatment during differentiation (shown as days) in cohort 2. Each circle represents one animal, color-coded to represent myeloid (M), myelo-lymphoid (ML), erythro-myeloid (EM) and erythro-myelo-lymphoid (MLE) patterns of engraftment. The number of mice receiving each duration of RETA (*n*) is shown. The number of unengrafted (NEG) mice is indicated. Data from 4CH 3B5A and 4CH 30A mesoderm inductions were pooled because they functioned similarly in the cohort 1 transplant experiments (Fig. 2c). Error bars, mean ± s.e.m. **c**, Confocal images of BM cells from an engrafted (m536) and unengrafted (m534) recipient. Scale bar, 50 µm. **d**–**g**, Flow cytometry profiles from BM (**d**), peripheral blood (PB; **e**), spleen (SPL; **f**) and thymus (THY; **g**) of a multilineage repopulated recipient (m490). **d**, Erythroid cells (CD43^+^GYPA^+^) were enriched in the TOM low (lo) BM fraction. The TOM high (hi) BM cells comprised CD19^+^ B cells, CD33^+^/CD13^+^ myeloid cells and CD45^+^CD34^+^CD38^lo/−^ HSC-like cells (boxed in red). **f**, The SPL contained CD45^+^CD19^+^sIGM^+^ B cells. **g**, The THY contained immature CD45^+^CD3^−^ thymocytes including CD4^−^CD8^−^ cells, transitioning through immature CD4^+^ to CD4^+^CD8^+^ double-positive cell states, whilst CD45^+^CD3^+^ thymocytes included CD4^+^CD8^+^ double-positive and CD4^+^ and CD8^+^ single-positive cells. Source data

The ACTINN analysis identified the association of a longer duration of RETA treatment with a greater proportion of *HLF*^+^*SPINK2*^+^ iPS cell-derived cells that mapped to CS14–15 AGM HSPCs. We explored the functional ramifications of this observation by varying the duration of retinoid exposure in a second series of transplantation experiments. In ten experiments using cells sourced from six independent differentiations, 103 animals (cohort 2) were injected with differentiated RM TOM hematopoietic cells exposed to increasing durations of retinoid treatment (screening protocol 2 in Extended Data Fig. 1a, Fig. 4a,b and Supplementary Tables 2, 3 and 11). Data from mice receiving cells in which mesoderm was induced with 4CH 3B5A or 4CH 30A were pooled, given that both variations displayed a similar expression of HSC signature genes (Extended Data Fig. 2a). MLE was seen in 6/25 (24%) mice transplanted with cells treated with RETA from days 3 to 5, the duration of RETA that was successful for engraftment in cohort 1, and in 7/19 (36.8%) mice receiving cells exposed to RETA treatment from days 3 to 13, although the difference did not achieve statistical significance. We did not see MLE in mice receiving cells treated with RETA from days 3 to 7 or 9 and only in one of eight mice receiving RETA from days 3 to 15 of differentiation (Fig. 4b). While this suggests that retinoid signaling in hematopoiesis is temporally tightly regulated, we are cautious about overinterpreting these results because they were based on small numbers of animals transplanted with cells derived from only two differentiation experiments (Supplementary Table 11). All mice with MLE were analyzed >16 weeks after transplantation, with one exception (analyzed at 15.7 weeks). Notably, these experiments confirmed that prolonged treatment with RETA was compatible with the in vitro generation of MLE iHSCs from iPS cells, consistent with our transcriptomic data showing that prolonged exposure to a retinoic acid precursor was required for the expression of retinoid-responsive genes (Extended Data Fig. 2d) and embryo data indicating that AGM-derived HSCs develop in a retinoid-conditioned milieu^13,20^ (Supplementary Fig. 2c).

**Extended Data Fig. 5:**
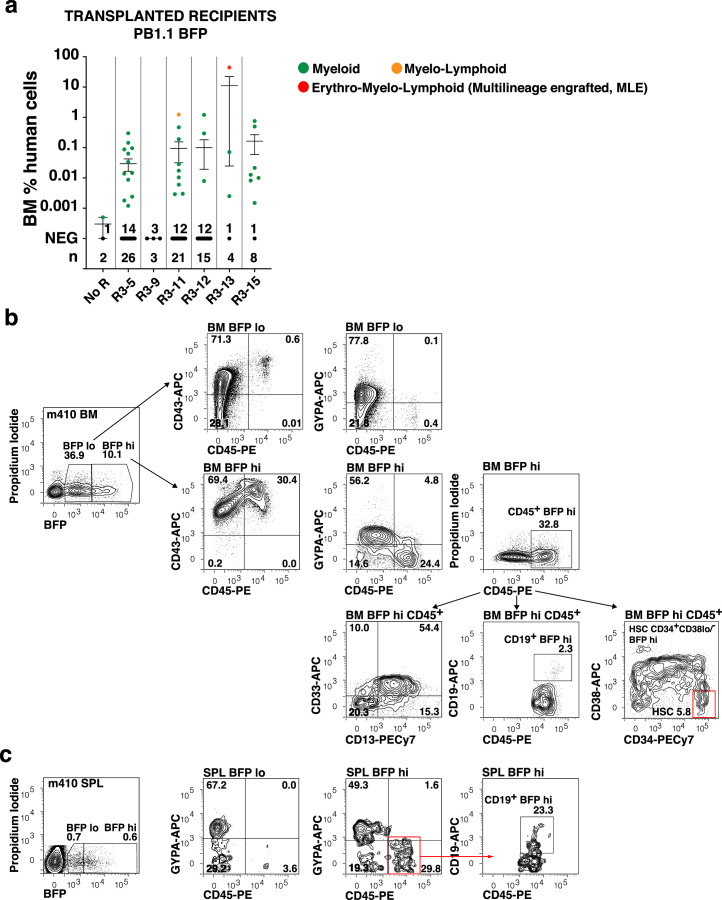
Engraftment of blood cells from PB1.1 BFP iPSCs in cohort 3 transplant recipients. (**a**) Scatter dot plot correlating human cells in the bone marrow (BM) with the interval of retinoid (R) treatment during differentiation (shown as days). Each circle represents one animal, colour coded to represent myeloid, myelo-lymphoid, and erythro-myelo-lymphoid multilineage (MLE) patterns of engraftment. Number of mice receiving each duration of retinoid during differentiation (n) is shown. Number of unengrafted (NEG) mice indicated. Error bars, mean ± s.e.m. Data from 4CH 3B5A and 4CH 30 A mesoderm inductions was pooled. Flow cytometry profiles from (**b**) bone marrow (BM) and (**c**) spleen (SPL) of a multilineage repopulated recipient (mouse (m)410). (**b**) Erythroid cells (GYPA^+^CD43^+^) were enriched in the BFP low (lo) BM fraction. BFP high (hi) cells included erythroid cells (GYPA^+^ CD45^−^), CD19^+^ B cells, CD33^+^ and CD13^+^ myeloid cells, and CD45^+^CD34^+^CD38^lo/−^ HSCs. (**c**) The spleen also contained erythroid cells (GYPA^+^CD45^-^) and CD19^+^CD45^+^ B cells. Source data

We performed a similar series of experiments using the second transcriptionally profiled human iPS cell line, PB1.1 BFP, transplanting 79 mice in eight experiments derived from six independent differentiation experiments (cohort 3). Bone marrow engraftment was observed in 44.3% of recipients with predominantly myeloid-restricted engraftment, although one mouse demonstrated MLE after 19 weeks (Extended Data Fig. 5a–c and Supplementary Table 12). These data demonstrated that our differentiation protocol enabled the generation of MLE cells from a second independent iPS cell line, although the lower frequency of engraftment highlighted the requirement for protocol improvement.

### MLE recipients of iHSCs showed MLE of hematopoietic tissues and establishment of a bone marrow stem cell compartment

**Extended Data Fig. 6:**
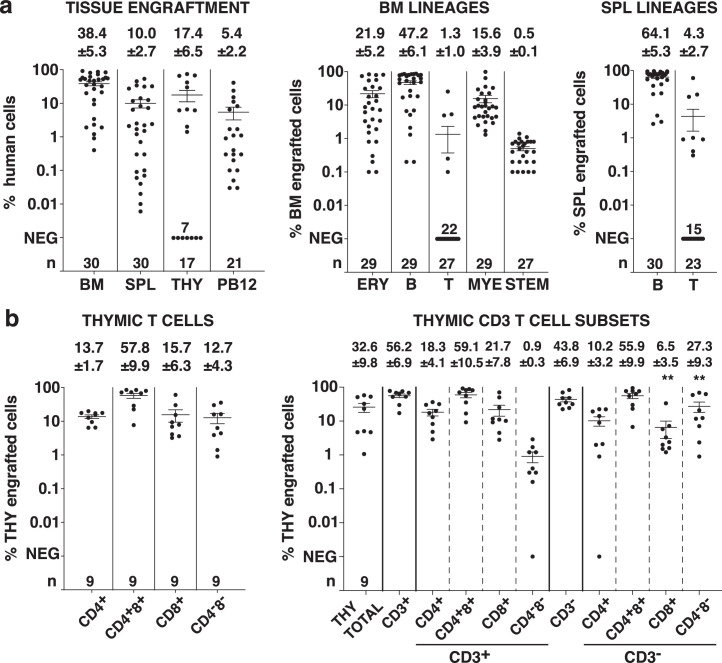
Tissue distribution and lineages in cohort 1–3 multilineage engrafted recipients of iHSCs. (**a**) Left panel, tissue engraftment showing human cells in bone marrow (BM), spleen (SPL), thymus (THY) and peripheral blood at 12 weeks (PB12). Middle and right panels, lineage distribution in the BM and SPL of reconstituted mice. ERY, erythroid; B, B cell; T, T cell; MYE, myeloid; STEM, hematopoietic stem and progenitor cells. Error bars, mean ± s.e.m. (**b**) Left panel, major thymic T cell subset distribution. Right panel, distribution of T cell subsets in CD3^+^ and CD3^−^ thymocytes. Statistics, CD8^+^CD3^+^ vs CD8^+^CD3^−^, ** *P* = 0.0039, two-tailed Wilcoxon matched-pairs signed rank test. CD4^-^CD8^−^CD3^+^ vs CD4^−^CD8^−^CD3^−^, ** *P* = 0.0078, two-tailed Wilcoxon matched-pairs signed rank test. Error bars, mean ± s.e.m. Source data

We examined the contribution and lineage distribution of human cells in the bone marrow, spleen, thymus and peripheral blood of MLE animals identified in cohorts 1–3 in more detail (Fig. 4c–g and Extended Data Fig. 6). Human cells were present in the peripheral blood at 12 weeks after transplantation in MLE recipients (Extended Data Fig. 6a and Supplementary Table 13). Confocal analysis showed readily observable TOM^+^ human cells in the bone marrow (Fig. 4c), whilst flow cytometry analysis revealed the presence of erythroid, myeloid and B lymphoid cells in the bone marrow, as well as splenic B and T cells and, in some animals, developing thymic T cells (Fig. 4d–g, Extended Data Figs. 5b and 6a,b and Supplementary Table 13). Immature CD3^−^ thymocytes passed from the CD4^−^CD8^−^ stage through an intermediate single-positive CD4^+^ stage to CD4^+^CD8^+^ double-positive thymocytes and CD3^+^ double-positive thymocytes gave rise to single-positive CD4^+^ and CD8^+^ T cells (Fig. 4g, Extended Data Fig. 6b and Supplementary Table 13). Erythroid cells in the bone marrow stained for cell-surface GYPA and CD43 and predominantly expressed low levels of the TOM or BFP reporter genes (Fig. 4d and Extended Data Fig. 5b). This was consistent with prior observations that maturing erythroid cells preferentially transcribed globin genes and reduced expression from the *GAPDH* locus^28,29^. Another defining characteristic of the MLE animals was the presence of a bone marrow CD45^+^CD34^+^CD38^lo/−^ HSC-like population (Fig. 4d, Extended Data Figs. 5b and 6a and Supplementary Table 13).

### Modulating VEGF signaling enhances MLE in recipients from multiple independent iPS cell lines

**Extended Data Fig. 7:**
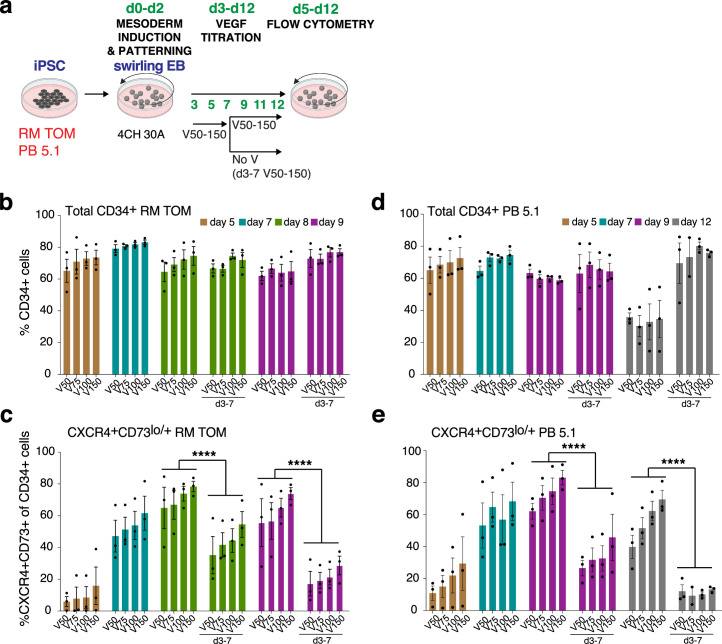
Removal of VEGF at day 7 of differentiation accelerates the loss of arterial endothelial markers. (**a**) Swirling embryoid body (EB) differentiation protocol outlining the VEGF (V) titration. Numbers represent VEGF concentration in ng ml^-1^. Partially created using BioRender.com. (**b**) CD34 expression in RM TOM cells by flow cytometry correlated with VEGF concentration. Error bars, mean ± s.e.m., n = 3. One-way ANOVA test for CD34 linear trend with VEGF, *P* < 0.0001. (**c**) Percentage of RM TOM CXCR4^+^CD73^lo/+^ arterial cells, subsetted from CD34^+^ cells, by flow cytometry correlated with VEGF concentration. Error bars, mean ± SEM, n = 3. One-way ANOVA test for CXCR4^+^CD73^lo/+^ linear trend with VEGF, *P* < 0.0001. Comparison of d8 and d9 samples continuing VEGF with d3-7 VEGF, both *P* < 0.0001, one-way ANOVA with Sidak’s multiple comparisons test. (**d**) CD34 expression in PB5.1 cells by flow cytometry correlated with VEGF concentration. Error bars, mean ± s.e.m., n = 3. One-way ANOVA test for CD34 linear trend with VEGF for day 5 and day 7, *P* = 0.0105. (**e**) Percentage of PB5.1 CXCR4^+^CD73^lo/+^ arterial cells, subsetted from CD34^+^ cells, by flow cytometry correlated with VEGF concentration. Error bars, mean ± s.e.m., n = 3. One-way ANOVA test for CXCR4^+^CD73^lo/+^ linear trend with VEGF, *P* < 0.0001. Comparison of d9 and d12 samples continuing VEGF with d3-7 VEGF, both *P* < 0.0001, one-way ANOVA with Sidak’s multiple comparisons test. Source data

**Extended Data Fig. 8:**
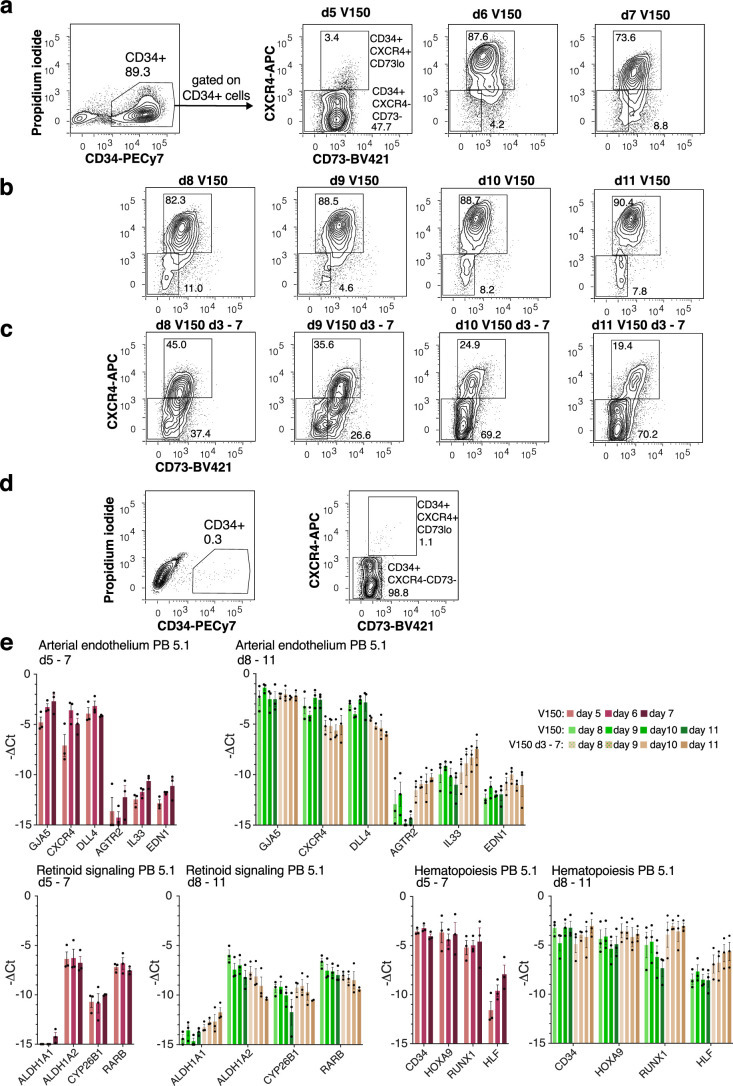
Removal of VEGF at day 7 of differentiation increases expression of aortic pre-hemogenic endothelial genes. (**a**) Flow cytometry analysis of differentiating PB5.1 cells showing the increase in CD34^+^CXCR4^+^CD73^lo/+^ arterial cells in response to VEGF at 150 ng ml^-1^ (V150) from d3–7. (**b**) Continuing VEGF maintains CXCR4 expression. (**c**) VEGF removal leads to CXCR4 downregulation. (**d**) Negative control samples unstained for CD34 or for CXCR4 and CD73. (**e**) Real time PCR analysis of differentiated samples of PB5.1 analyzed from d5–11 for the indicated arterial endothelium, retinoid signaling and hematopoietic genes. Samples with continued VEGF signaling are compared to samples where the VEGF was removed after d7. Error bars, mean ± s.e.m., n = 3 independent experiments. V150 vs V150 d3-7 at d11: *AGTR2*, *P* = 0.0125; *IL33*, *P* = 0.0307; *RUNX1*, *P* = 0.0009; *HLF*, *P* = 0.0100; mixed-effects analysis (two-way ANOVA) with Sidak’s multiple comparisons test. Source data

The lack of efficient generation of iHSCs from the PB1.1 BFP cell line in cohort 3 mice led us to consider modifications to the protocol growth factor composition that might improve the robustness of hematopoietic differentiation. Evidence from the human embryo^13^ suggests that HSCs arise from an arterially patterned hemogenic endothelium. VEGF acts in a dose-dependent manner to drive endothelium generation and arterialization in differentiating PS cells^30–33^. However, recently published work using a murine embryonic stem cell differentiation system showed that VEGF suppressed hematopoietic progenitor development from endothelium by blocking the upregulation of *Runx1* expression^21^, a critical marker of hemogenic endothelium and regulator of HSC development in the mammalian embryo^13,34^. To explore these opposing effects, we trialed a range of VEGF concentrations from days 3 to 7, during endothelial generation, followed by continuing or removing VEGF to determine which best enhanced the endothelial-to-hematopoietic transition. We demonstrated a VEGF dose-dependent increase in CD34^+^CXCR4^+^ arterial endothelial cell generation, followed by a rapid loss of the arterial marker CXCR4 after the removal of VEGF on day 7 of differentiation (Extended Data Fig. 7). Gene expression analysis revealed that the combination of high VEGF from day 3 followed by its removal on day 7 of differentiation increased the expression of aortic endothelial genes (*AGTR2*, *IL33* and *EDN1*), reduced *ALDH1A2* and increased *ALDH1A1*, accelerated the endothelial-to-hematopoietic transition, evidenced by the reduction in *CXCR4* and *DLL4*, and increased *RUNX1* and *HLF* expression (Extended Data Fig. 8). We previously identified many of these genes as being more lowly expressed in iPS cell-derived cells compared to the human embryo^13^ in an endothelial-to-hematopoietic transition scorecard (Extended Data Fig. 4f).

**Fig. 5:**
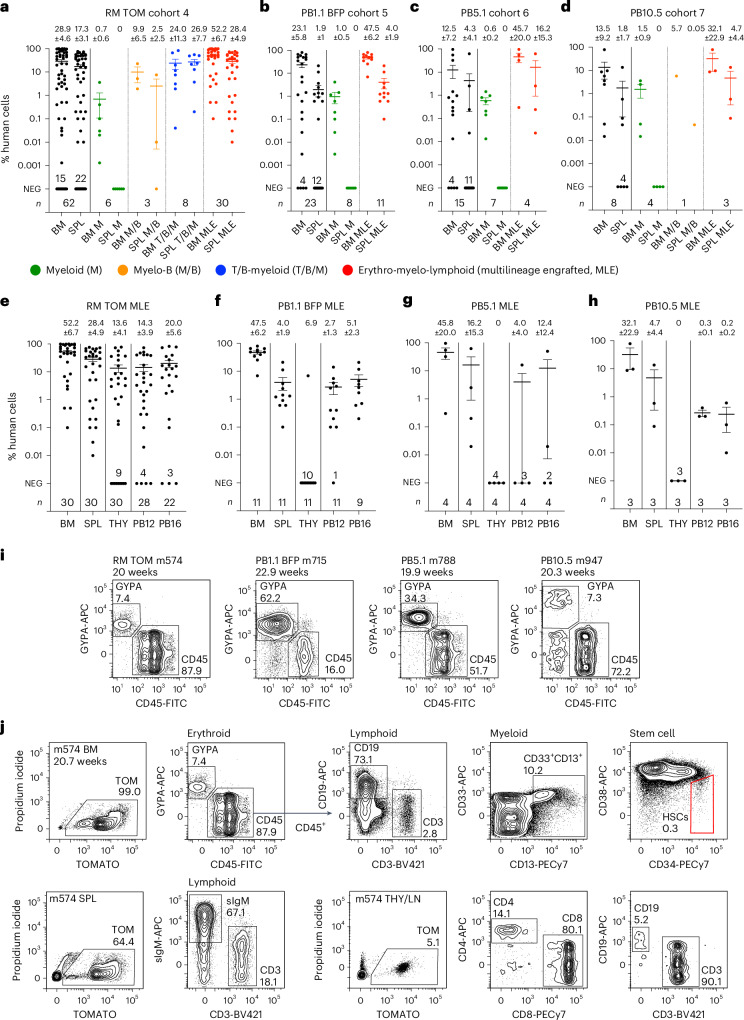
Robust hematopoietic engraftment with cells differentiated using protocol 3. **a**–**d**, Engraftment of BM and SPL in transplant recipients of RM TOM (**a**), PB1.1 BFP (**b**), PB5.1 (**c**) and PB10.5 (**d**) cells showing the phenotype of engrafting cells and the level of engraftment. Error bars, mean ± s.e.m. **e**–**h**, Tissue distribution of engrafting cells in MLE recipients of RM TOM (**e**), PB1.1 BFP (**f**), PB5.1 (**g**) and PB10.5 (**h**) cells in BM, SPL, THY and PB at 12 and 16 weeks. Error bars, mean ± s.e.m. **i**, Flow cytometry analysis of BM in engrafted mice for each cell line showing GYPA^+^ erythroid lineage and CD45^+^ lymphoid and myeloid cells. **j**, BM, SPL and THY or mediastinal lymph node (LN) tissue of RM TOM-engrafted mouse m574, showing GYPA^+^ erythroid, CD45^+^CD19^+^ B cell, CD45^+^CD3^+^ T cell, CD45^+^CD33^+^/CD13^+^ myeloid and CD45^+^CD34^+^CD38^lo/-^ stem cell populations in the BM, CD45^+^sIgM^+^ B cells and CD45^+^CD3^+^ T cells in the SPL and THY or mediastinal LN tissue containing CD45^+^CD3^+^CD4^+^ and CD45^+^CD3^+^CD8^+^ T cells and a population of CD45^+^CD19^+^ B cells. Source data

We incorporated these modifications into the next evolution of the differentiation protocol (denoted protocol 3; Extended Data Fig. 1a) and explored their functional consequences in further transplantation experiments. In mice transplanted with RM TOM cells (cohort 4), we observed improved engraftment compared to the earlier experiments (cohorts 1 and 2), recording 30/62 (48.4%) mice with MLE, with 61/62 recipients analyzed >16 weeks after transplantation (Fig. 5a and Supplementary Tables 2, 3 and 14). Similar engraftment results were seen in three additional human iPS cell lines, including the PB1.1 BFP line that transplanted less efficiently in experiments (cohort 3) using the previous differentiation protocol (Extended Data Fig. 1a). MLE was observed in 11/23 (47.8%) of PB1.1 BFP (cohort 5), 4/15 (26.7%) of PB5.1 (cohort 6) and 3/8 (37.5%) of PB10.5 (cohort 7) mice, analyzed >16 weeks after transplantation in 41/46 cases (Fig. 5b–d and Supplementary Tables 2, 3 and 15–17). These results indicated that protocol 3 generated more robustly engrafting cells and was applicable to a broader range of iPS cell lines. We have not yet performed limit dilution transplantation experiments using this protocol but analysis of the overall engraftment results given above suggests that the frequency of MLE cells was 1 in 3.0 × 10^6^ for the RM TOM, 1 in 3.1 × 10^6^ for the PB1.1 BFP, 1 in 6.2 × 10^6^ for the PB5.1 and 1 in 4.3 × 10^6^ for the PB10.5 lines^35^. We found some variability in outcomes between experiments, with estimated engraftment frequencies as high as 1 in 1.3 × 10^6^ for RM TOM experiment E427 in which 7/9 recipients displayed MLE (Supplementary Tables 14–17). In summary, these results still show variation in engraftment between different lines and between experiments. We believe that this variability may be lessened with further protocol optimization.

**Extended Data Fig. 9:**
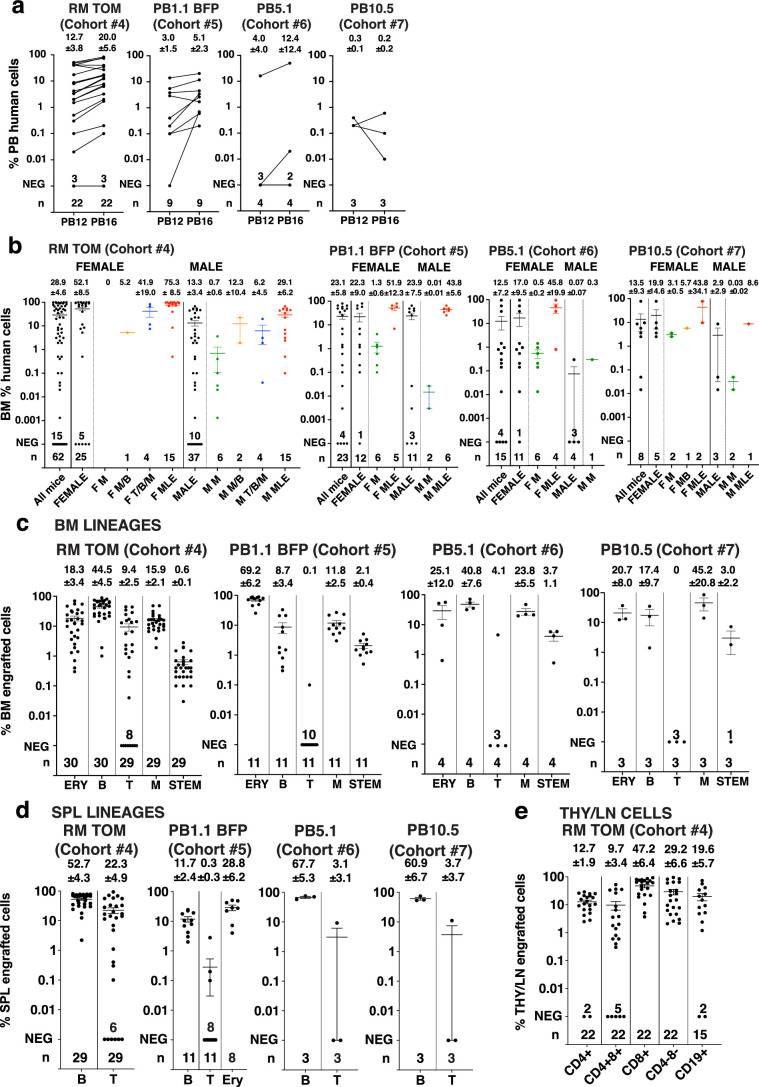
Contribution and lineage distribution of human cells in the bone marrow, spleen, thymus and peripheral blood of cohort 4–7 mice receiving cells differentiated under protocol 3. (**a**) Paired samples of peripheral blood analyzed at 12 (PB12) and 16 weeks (PB16) in MLE mice. (**b**) Bone marrow engraftment in transplanted mice stratified by recipient sex. RM TOM cells, Female vs Male mice, *P* = 0.0012; Female MLE vs Male MLE, *P* = 0.0427; PB5.1, Female vs Male mice, *P* = 0.0253; PB1.1 BFP and PB10.5, no significant gender differences. One-way ANOVA (Kruskal-Wallis test) with Dunn’s multiple comparisons test. (**c**) Bone marrow and (**d**) spleen lineage distribution in MLE mice in cohort 4–7. (**e**) T cell subsets and B cells in mediastinal lymphoid tissue, comprising thymus and lymph node tissue in MLE mice in cohort 4. Source data

We analyzed the contribution and lineage distribution of human cells in the bone marrow, spleen, thymus and peripheral blood of the 48 MLE animals receiving cells differentiated under protocol 3 (Fig. 5e–h and Extended Data Fig. 9). In most cases, human cells were present in the peripheral blood at 12 weeks after transplantation (38/46 mice analyzed) (Fig. 5e–h) and the evaluation of paired samples at 16 weeks revealed an increase in human cells in 28/35 mice analyzed across the four cell lines (Extended Data Fig. 9a). There was an evident sex bias in bone marrow engraftment, most prominent in the RM TOM line recipients (Extended Data Fig. 9b), with significantly higher levels of human cells in female than male recipients, consistent with the published literature^36^. Over all experiments (cohorts 1–7), MLE was seen in 19.6% of recipients transplanted with CD34^+^ suspension blood cells, 23.8% of those receiving CD34-enriched cells from the EBs and 24.5% of mice that received both suspension blood cells and CD34-enriched cells from the EBs. These proportions were not statistically different and demonstrated that stem cells were present in CD34^+^ cells from both sources (Supplementary Table 18).

Flow cytometry analysis revealed the presence of bone marrow erythroid, myeloid, B and T lymphoid cells and CD45^+^CD34^+^CD38^lo/−^ HSC-like cells, splenic B and T cells and thymic T cells, similar to MLE animals in cohorts 1–3 (Fig. 5i,j, Extended Data Fig. 9c–e and Supplementary Table 19). A comparison of mice engrafted with RM TOM cells under the different protocols revealed higher percentages of human cells in the bone marrow, spleen and peripheral blood in recipients of protocol 3 differentiated cells, with a persistent bias toward higher engraftment in female mice (Supplementary Fig. 4a,b). The proportions of erythroid, myeloid, B and stem cells were similar in male and female recipients but the proportion of T cells in the bone marrow and spleen were greater in engrafted female mice (Supplementary Fig. 4c,d).

**Fig. 6:**
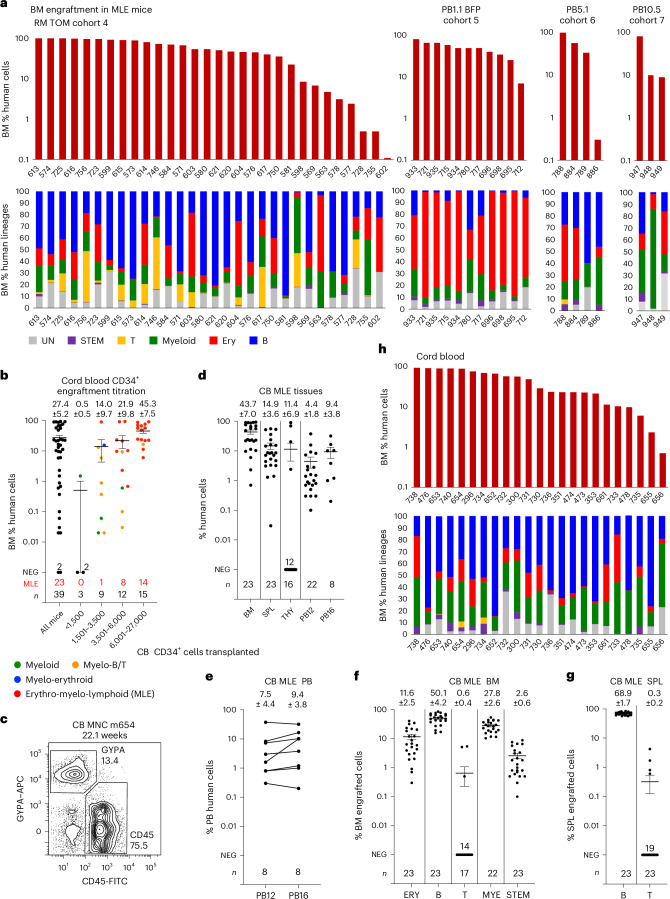
Engraftment patterns of MLE iHSC and CB transplanted mice. **a**, Top: bar graphs showing the level of human engraftment in the BM of MLE mice receiving the indicated cell lines (individual recipients identified on *x* axis). Bottom: stacked column graphs showing the lineage distribution of human cells in the BM of iHSC-engrafted recipients. UN, unclassified cells include myeloid, dendritic and natural killer cells not detected by the antibodies used (Supplementary Table 19). **b**–**h**, Characteristics of engrafted CB cells. **b**, Scatter plot correlating calculated dose of injected CD34^+^ CB cells with phenotype and level of human engraftment in the BM. Each circle represents one animal, color-coded to represent M, ML, ME and MLE patterns of engraftment. Error bars, mean ± s.e.m. A total of 39 animals were transplanted. **c**, Flow cytometry plot showing GYPA^+^ erythroid cells and CD45^+^ lymphoid and myeloid cells. **d**, Tissue distribution of engrafting cells in MLE recipients of CB cells in BM, SPL, THY and PB at and 16 weeks. **e**, Analysis of paired samples of PB showing increased levels of human cells in 6/8 recipients between 12 and 16 weeks. **f**,**g**, Lineage distribution in the BM (**f**) and SPL (**g**) in CB recipients. **h**, Top, bar graphs showing the level of human engraftment in BM of MLE mice receiving CB cells (individual recipients identified on *x* axis). Bottom, stacked column graphs showing the lineage distribution of human cells in the BM of CB-engrafted recipients. UN, unclassified cells include myeloid, dendritic and natural killer cells not detected by the antibodies used (Supplementary Table 21). Source data

**Extended Data Fig. 10:**
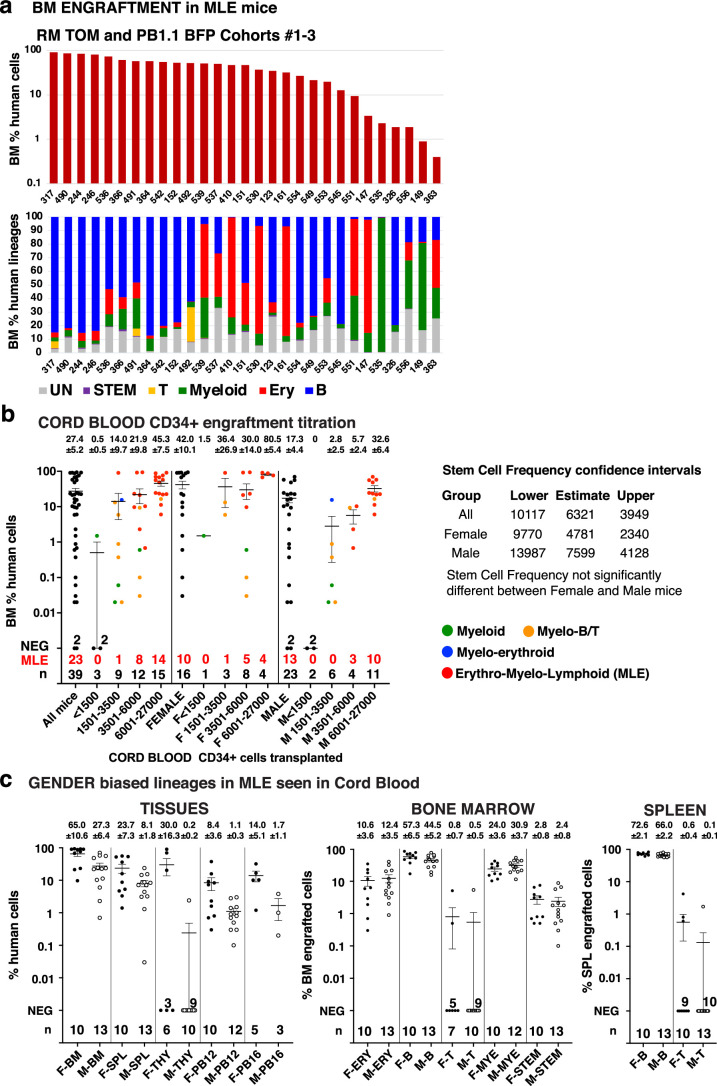
Engraftment patterns of MLE engrafted iHSC and CB transplanted mice. (**a**) Top, bar graphs show the level of human engraftment in BM of MLE mice receiving RM TOM and PB1.1 BFP lines in cohort 1-3 transplants (individual recipients identified on x-axis). Bottom, stacked column graphs showing the lineage distribution of human cells in the BM of iHSC engrafted recipients. UN, unclassified cells include myeloid, dendritic and natural killer cells not detected by the antibodies used. See also Supplementary Table 13. (**b**, **c**) Characteristics of engrafted CB cells. (**b**) Scatter plot correlating calculated dose of injected CD34^+^ CB cells with phenotype and level of human engraftment in the bone marrow, with results stratified by recipient gender. Each circle represents one animal, color coded to represent myeloid, myelo-lymphoid, myelo-erythroid and erythro-myelo-lymphoid multilineage (MLE) patterns of engraftment. Error bars, mean ± s.e.m. Total of 39 animals transplanted. Stem cell frequency was estimated by limit dilution assay^35^. (**c**) Tissue distribution, bone marrow and spleen lineages of engrafting cells in MLE recipients of CB cells stratified by recipient gender. Female recipients displayed higher levels of bone marrow (*P* = 0.0117), thymus (*P* = 0.0357), and peripheral blood engraftment at 12 weeks (*P* = 0.0340). Mann-Whitney t-tests. Source data

Where T cell development was observed in the spleen and bone marrow, we rarely observed a macroscopically identifiable bilobed thymus but small amounts of putative lymphoid tissue were frequently present in the mediastinum. This tissue usually contained single-positive CD4^+^ and CD8^+^ cells, together with few double-positive thymic cells and often a population of CD19^+^ B cells (Fig. 5j, Extended Data Fig. 9e and Supplementary Table 19). The low percentage of CD4^+^CD8^+^ cells was particularly marked in the more highly engrafted cohort 4 female (1.8% ± 0.7% CD4^+^CD8^+^ cells) compared to male (23.7% ± 6.9% CD4^+^CD8^+^ cells) mice receiving cells differentiated using protocol 3 and contrasted with the high proportion of CD4^+^CD8^+^ cells seen in female (52.7% ± 12.1%) and male (75.7% ± 4.1%) mice receiving protocol 1 and 2 differentiated cells (Supplementary Fig. 4c,d and Supplementary Table 20). We speculate that this result might reflect an inability to sustain thymic tissue in aging immune-deficient mice, with also likely sampling of mediastinal lymph nodes to account for the presence of B cells (Fig. 5j and Supplementary Tables 19 and 20). These observations may have been more prominent in cohort 4 recipients because the degree of T cell engraftment, evident by T cell contribution to the human cells in the bone marrow and spleen, was greater in recipients of protocol 3 differentiated cells (compare cohort 4 recipients in Fig. 6a to cohort 1–3 recipients in Extended Data Fig. 10a). A similar dimorphic pattern of T cell engraftment was observed in immune-deficient mouse recipients of CB CD34^+^ cells, in which a major CD4^+^CD8^+^ thymic population was seen in 10/19 mice with T cell engraftment, while low CD4^+^CD8^+^ cell numbers were seen in the remainder^37^.

### Heterogeneity of lineage composition in MLE recipients of iHSCs

Despite robust overall transplantation of human cells, lineage contributions varied among different MLE mice and recipients of cells differentiated from independent iPS cell lines (Extended Data Figs. 6 and 9 and Supplementary Tables 13 and 19). The bone marrow of most mice receiving RM TOM cells in cohorts 1–3 was dominated by B cells (Extended Data Fig. 10a). Some mice showed predominantly erythroid engraftment and the cohort 4 mice receiving cells differentiated under protocol 3 also frequently displayed T cell engraftment (Fig. 6a). Recipients of PB1.1 BFP differentiated cells (cohort 5) displayed dominant erythroid engraftment, whilst the smaller number of recipients of PB5.1 (cohort 6) and PB10.5 (cohort 7) lines showed more balanced engraftment patterns (Fig. 6a, Extended Data Fig. 9 and Supplementary Table 19). We observed that engraftment was maintained in the bone marrow and spleen in animals evaluated for >16 weeks, consistent with stable engraftment by long-term repopulating cells (Supplementary Fig. 5).

### Umbilical CB mononuclear cells display dose-dependent engraftment

We wished to provide a relevant context for our experiments by comparing the engraftment phenotypes of iPS cell-derived iHSCs to those of CB cells, a clinically validated source of repopulating HSCs. A total of 39 mice were transplanted with 5 × 10^4^–2.5 × 10^6^ CB mononuclear cells isolated from four separate cords that comprised 0.7–2.7% CD34^+^ cells, resulting in the transplantation of 3.5 × 10^2^–2.7 × 10^4^ CD34^+^ cells. MLE was observed in most recipients (14/15) of mononuclear cells calculated to contain > 6.0 × 10^3^ CD34^+^ cells (Fig. 6b and Supplementary Table 21) and the estimated frequency of repopulating CB stem cells was 1 in 6.3 × 10^3^ CD34^+^ cells according to a limit dilution assay^35^ (Extended Data Fig. 10b), consistent with reports in the literature^37^. Similar to our findings with iPS cell-derived iHSC transplants, CB cells showed higher levels of engraftment in female mice (Extended Data Fig. 10b,c). Mice receiving fewer than 6.0 × 10^3^ CB CD34^+^ cells showed lower total proportions of human cells in the bone marrow. Moreover, they frequently displayed restricted lineage engraftment with myeloid or myeloid and lymphoid lineages (Fig. 6b). This positive correlation between engraftment level and MLE in recipients of CB stem cells mirrored the similar correlation observed in the iPS cell-derived blood cell transplants (Supplementary Results 2). This observation also aligned with reported dose-dependent hematopoietic chimerism in immune-deficient mice receiving purified CB stem cells, where engraftment with low stem cell numbers similarly led to low levels of myeloid or myeloid-restricted and B cell-restricted engraftment that persisted for 19–21 weeks^37^. Taken together, this indicated that the transplantation assay reads out a hierarchy of stem cells for both CB-derived and iPS cell-derived cells. MLE cells with high proliferative capacity are less abundant than myeloid or myeloid-restricted and lymphoid-restricted stem cells with low proliferative capacity.

The profile of engrafted lineages was similar between CB and iHSC recipients, although T cell engraftment was greater in the RM TOM mice (compare Fig. 6c–g to Extended Data Fig. 9a–d). PB1.1 BFP recipients displayed prominent erythroid engraftment in the bone marrow and spleen, with commensurately lower levels of lymphoid and myeloid engraftment (Extended Data Fig. 9c–d). In CB recipients, the most abundant lineages were B and myeloid cells, with few mice displaying large erythroid populations and few cases of T cell engraftment. Heterogeneity in the distribution of bone marrow lineages in individual MLE CB mice can be appreciated in the bar graphs in Fig. 6h and compared to similar data for iHSCs shown in Fig. 6a and Extended Data Fig. 10a.

### iHSCs show comparable secondary engraftment to CB HSCs

We investigated whether bone marrow cells from primary recipients engrafted with either CB-derived or iPS cell-derived HSCs could engraft secondary recipients. We observed secondary engraftment from 6/12 primary mice engrafted with iHSCs and from 2/5 primary mice engrafted with CB cells, with similar outcomes observed from primary recipients engrafted with cells generated by different protocols (Discussion, Supplementary Discussion and Supplementary Table 22). In most cases, engraftment was at a low level and restricted to myeloid lineages, although one iHSC secondary transplant recipient displayed B, T and myeloid lineages in the bone marrow, spleen and thymus.

## Discussion

This work describes a method that generates repopulating iHSCs from human iPS cells and demonstrates that they display characteristics of primary HSCs or multipotent progenitors, evidenced by transcriptional similarity to HSPCs from the CS14 embryonic AGM region^13^, the high-level, long-term erythroid, myeloid and lymphoid engraftment of immune-deficient mice and the establishment of bone marrow HSC-like cells similar to those seen in mice engrafted with CB and AGM^12,38^. The reported frequency of engrafting CD34^+^ cells in granulocyte colony-stimulating factor-mobilized peripheral blood^39^, the most common source of stem cells for transplantation, was only 20-fold higher than that of iHSCs. We expect that the proportion of MLE iHSCs generated in our cultures can be increased through ongoing improvements to the differentiation protocol, combined with the inclusion of strategies to maintain HSC function, such as those recently reported to expand human CB HSCs^40^.

Low levels of secondary engraftment were observed following serial transplantation, despite the presence of cells with a CD34^+^CD38^lo/−^ stem cell-like phenotype in the bone marrows of MLE recipients. We believe that this reflected the small amounts of primary bone marrow transplanted (0.3–2.0 × 10^6^ bone marrow cells), combined with the suboptimal niche provided by the NBSGW mouse bone marrow environment^38,41^. The observation that similarly low levels of secondary engraftment were seen with bone marrow cells from primary recipients of CB cells indicated that this was not a finding restricted to iPS cell-derived hematopoietic cells. Indeed, low levels of secondary engraftment have been reported in several studies of immune-deficient mice transplanted with human CB^24,37,38^. Furthermore, similar to our results, in another study in which CD34^+^ CB cells engrafted into NBSGW mice were secondarily transplanted, low levels of myeloid lineage-restricted secondary engraftment were seen despite each recipient receiving 3 × 10^7^ bone marrow cells^41^. However, a study from the Medvinsky laboratory using NOD *scid* gamma (NSG) immune-deficient mice^38^ showed that secondary transplantation with 7.5 × 10^6^ CB-engrafted bone marrow cells gave peripheral blood engraftment of 0.5–7.3% human cells at 5 months. While engraftment levels were still low, the result suggested that the NSG mouse may be superior to NBSGW in maintaining functional stem cells. Furthermore, in the same study, bone marrow from mice engrafted with human AGM-derived HSCs produced secondary engraftment in animals transplanted with as few as 5 × 10^5^ bone marrow cells^38^. With the caveat that our study and that of Medvinsky used different strains of immune-deficient mice, transplantation of cells from iHSC-engrafted bone marrow might have been expected to result in higher proportions of secondarily engrafted recipients if the iHSCs displayed the same degree of self-renewal as true AGM-derived HSCs. Our results suggest that iHSCs generate a bone marrow HSC compartment with similarly functioning stem cells to CB but may lack the ability for substantial expansion that marks AGM-derived HSCs. This question may be resolved by future studies that use a different immune-deficient mouse strain (such as NSG) and larger numbers of bone marrow cells to determine whether robust secondary engraftment is observed from primary iHSC-engrafted mice.

Dissecting the contribution of different growth factors in our cultures to the generation of MLE cells under protocol 3 demonstrated that this outcome was contingent on the sequential induction of mesoderm by CHIR and a transforming growth factor-β family member, its patterning to *HOXA* positivity by a Wnt agonist and ALK antagonist, arterial hemogenic endothelium formation by BMP4, a high concentration of VEGF and a retinoic acid precursor and an enhancement of the endothelial-to-hematopoietic transition through the timely removal of VEGF. Retinoic acid signaling was required for the generation of AGM HSCs in the mouse^19,20^ and retinoid-responsive genes are expressed in human AGM cells^13,18^. It is notable that the proportion of cells in our cultures transcriptionally similar to CS14–15 AGM HSPCs increased in response to retinoid inclusion and correlated with the emergence of MLE cells. A recent study identified a retinoic acid-responsive mesoderm fraction in day 3 differentiating human PS cells that developed into *HOXA*^+^ multilineage hematopoietic cells. However, unlike iHSCs, these cells were capable of only low-level, short-term engraftment in immune-deficient neonatal mice following intrahepatic injection^42^. Taken in the context of our work, the lack of long-term engraftment in this previous study highlights that retinoid signaling is necessary but not sufficient to confer high-level multilineage repopulation potential on iPS cell-derived blood cells. In a refinement of our protocol, we increased the concentration of VEGF during the generation of arterially patterned hemogenic endothelium and then completely removed it on the basis of recent observations that VEGF signaling can inhibit the expression of *RUNX1* and other downstream key hematopoietic genes^21,43^. These modifications enhanced the endothelial-to-hematopoietic transition and notably increased the robustness of engraftment.

Prior studies reporting long-term engraftment of iPS cell-derived hematopoietic cells required enforced expression of multiple transcription factors, which confirmed the importance of *HOXA* gene expression for the generation of HSC-like blood cells. One study reported engraftment of up to 30% human cells in the bone marrow assessed 12 weeks after transplantation in immune-deficient mice with human PS cell-derived cells expressing seven inducible transcription factors from lentiviral vectors (*ERG*, *HOXA5*, *HOXA9*, *HOXA10*, *LCOR*, *RUNX1* and *SPI1*)^44^. MLE was seen in ~15% of recipients and, in the absence of transcription factor induction, engraftment was not observed^44^. More recently, mouse embryonic stem cell-derived HSPCs formed following the forced expression of *Runx1*, *Hoxa9* and *Hoxa10* engrafted and persisted in recipient mice for up to 6 months^45^. The maximum embryonic stem cell-derived contribution to the bone marrow appeared to be under 30% and the frequency of donor cells in the peripheral blood diminished after 4 weeks. In contrast to these studies, MLE in our experiments was achieved without the expression of lentivirally introduced transgenes. We observed 25–50% human cells in the bone marrow of recipient mice across four iPS cell lines, with over 70% human cells in the bone marrow in 19/42 female recipients analyzed after 16 weeks. While some heterogeneity between genetically different iPS cell lines was observed with regard to the lineage output, our method permitted robust engraftment for all of them. Moreover, MLE mice displayed an increasing proportion of human cells in the peripheral blood between 12 and 16 weeks and sustained engraftment up to 24 weeks, the latest time of analysis.

Recently, Piau et al.^46^ differentiated iPS cells in a human plasma-containing medium supplemented with fixed concentrations of ten growth factors. They generated EBs that transcriptionally resembled a ‘teratoma in a dish’, containing ectodermal, mesodermal, endodermal and trophoblast lineage cells. Following intravenous transplantation of 4 × 10^5^ unsorted dissociated EB cells, MLE was reported in 59/60 immune-deficient recipients with bone marrow engraftment of 13.3% ± 1.5% human cells. The proportions of CD45^+^ and CD34^+^ cells in the EBs were low (<5%) and it was not proven that the initial engrafting cells were of hematopoietic origin. Secondary engraftment was seen in all 40 secondary recipients examined and levels of primary and secondary engraftment were similar to mice transplanted with a pure hematopoietic population of 4 × 10^5^ CD34^+^ CB cells. The protocol differed from ours with regard to (1) the absence of directed patterning to attempt to mimic normal hematopoietic development, (2) the exclusion of exogenously added retinoid or Wnt agonists; and (3) the inclusion of a chemically undefined plasma component. The lower level of bone marrow MLE compared to our study originated from a cell population that contained only rare cells expressing signature HSC genes, suggesting that the hematopoietic cells generated represented a different developmental stage than iHSCs. The work appears to resemble studies in which human iPS cell-derived teratomas developed repopulating HSCs. In one such study, CD34^+^ cells isolated from teratomas displayed myeloid-biased MLE with 1 × 10^4^ CD34^+^ teratoma cells yielding approximately 2.5% human cells in the bone marrow of immune-deficient mice^47^. A second study also detected human hematopoietic cells in teratoma-bearing mice that could be isolated and engrafted into immune-deficient recipients^48^. While previous studies^46–48^ demonstrated that repopulating HSCs can be derived from human iPS cells, their methods do not allow dissection of the mechanisms involved in patterning^49^ nor readily permit optimization to the cell numbers and purity required for engraftment in a clinical setting.

In summary, in this study, we showed that it is possible to use a fully defined culture system to differentiate human iPS cells in vitro to iHSCs that closely resemble the earliest HSCs in the human embryo. Injection of iHSCs into the tail vein of immune-deficient mice resulted in long-term MLE similar to that seen following transplantation with human CB. iHSCs could be cryopreserved before transplantation, recapitulating clinical HSC transplantation, which relies on cryopreserved donor hematopoietic cells. Thus, our method may enable the future generation of HSCs for clinical translation and disease modeling.

## Methods

### Ethics and inclusion

Local researchers were included throughout the research process and the local relevance of the research has been confirmed. Roles and responsibilities were agreed amongst collaborators ahead of the research and capacity-building plans for early-career local researchers were incorporated. Human PS cell studies were approved by The Royal Children’s Hospital Human Research Ethics Committee (reference 33001A). Samples of human umbilical CB from healthy subjects were obtained from the Bone Marrow Donor Institute (BMDI) CB Bank at the Murdoch Children’s Research Institute, under auspices of the The Royal Children’s Hospital Human Ethics Committee (reference 34170A, ID 42470). The Murdoch Children’s Research Institute animal ethics committee approved all animal protocols (reference A885 and A954). Citations to published work were based on scientific relevance, whether the research cited was local and regional or not.

### iPS cell culture and maintenance

RM TOM iPS cells, constitutively expressing a tdTOMATO transgene from the *GAPDH* locus, were derived from human foreskin fibroblasts purchased from the American Type Culture Collection and reprogrammed using the hSTEMCCAloxP four-factor lentiviral vector; integrated vector sequences were removed using Cre recombinase^23^. PB1.1 (male), PB10.5 (male) and PB5.1 (female) iPS cells were reprogrammed from the peripheral blood of healthy volunteers with Sendai virus carrying the reprogramming factors *POU5F1*, *SOX2*, *KLF4* and *MYC*^26^. PB1.1 was engineered to express mTagBFP2 from the *GAPDH* locus^23^. Following vector integration, Cre recombinase was used to excise the antibiotic selectable marker from this version of the targeting vector^50^. Human iPS cell lines were maintained by coculture with mouse embryo fibroblasts in KOSR medium (Thermo Fisher)^51^ for cells transplanted in cohort 1 and some cohort 2 experiments or adapted to culture on Matrigel (Corning) in Essential 8 medium (Thermo Fisher) for all experiments thereafter (cohorts 3–7). Molecular karyotyping by single-nucleotide polymorphism array was performed at regular intervals using the Illumina Infinium GSA-24 version 3.0 chip with a resolution of 0.50 Mb, with no clinically notable genomic imbalance detected. *Mycoplasma* contamination was excluded by regular testing.

### Harvest of iPS cells for initiation of differentiation

Hematopoietic differentiation was performed using the swirling EB method^13,22^. Cells were dissociated using Accutase cell dissociation reagent (Merck) and resuspended in SPELS differentiation medium, an evolution of APEL^52^ and STAPEL media^53^. SPELS medium includes nonessential amino acids, but not albumin or protein-free hybridoma medium (see Supplementary Table 23 for composition of SPELS medium). SPELS medium was supplemented during differentiation with growth factors as detailed below.

Approximately 2 × 10^6^ dissociated cells were transferred to each non-tissue-culture-treated 60-mm dish in 5 ml of SPELS medium. The dishes were placed on a digital orbital shaker (Heathrow Scientific) rotating at 60 r.p.m. in a 5% CO_2_ incubator at 37 °C.

### Identification of differentiation conditions that produce CD34^+^ hematopoietic cells with MLE ability: screening protocol 1 and mouse cohort 1

Versions of screening protocol 1 (encompassing 12 differentiation conditions) were analyzed in mouse cohort 1 transplantations to identify conditions that generated iHSCs. As shown diagrammatically (Extended Data Fig. 1a and Fig. 2a), the mesoderm was induced on day 0 of differentiation by a combination of 1, 2 or 4 µM CHIR99021 (Tocris Biosciences), 0 or 3 ng ml^−1^ recombinant human (rh) BMP4 (R&D Systems) and 5 or 30 ng ml^−1^ rh Activin A (R&D Systems). All conditions included 20 ng ml^−1^ rh FGF2 (PeproTech) and 1 µM Thiazovivin (Selleck Chem). Starting on day 1, medium changes occurred every 2 days throughout differentiation. From days 1–3, the mesoderm was patterned to *HOXA* expression with 3 µM CHIR99021 (Tocris Biosciences), 4 µM SB431542 (Cayman Chemicals or Selleck Chemical), 25 ng ml^−1^ rh VEGF (PeproTech), 25 ng ml^−1^ rh stem cell factor (SCF, PeproTech) and 20 ng ml^−1^ rh FGF2. On day 3, the medium was supplemented with 20 ng ml^−1^ rh BMP4, 50 ng ml^−1^ rh VEGF, 20 ng ml^−1^ rh FGF2, 50 ng ml^−1^ rh SCF and 10 ng ml^−1^ rh insulin-like growth factor 2 (IGF2, PeproTech). In selected transplantation experiments (Supplementary Tables 1, 11, 12 and 14–17), cultures were supplemented on day 3 of differentiation with 2 µM ROL or 2 µM RETA, which was removed during the day 5 medium change. From day 5 onward, rh BMP4 was reduced to 2 ng ml^−1^, while other growth factors were unchanged. In early experiments, 10 ng ml^−1^ APELIN peptide (Merck) was included from days 5 to 9. From day 11 of differentiation onward, growth factors included 50 ng ml^−1^ rh VEGF, 50 ng ml^−1^ rh SCF, 50 ng ml^−1^ rh thrombopoietin (TPO, PeproTech), 10 ng ml^−1^ rh FGF2 and 20 nM StemRegenin 1 (SR1, Selleck Chemical). Early experiments also included 10 ng ml^−1^ rh FLT3 receptor ligand (PeproTech) and 10 ng ml^−1^ rh interleukin 3 (PeproTech). Blood cells were shed into the medium after 10–12 days of differentiation. After days 14–16, cultures were harvested. Blood cells in the medium (suspension hematopoietic cells) were analyzed separately from cells dissociated from the swirling EBs, which were disaggregated by 45-min incubation with 2 mg ml^−1^ collagenase type I (Worthington) at 37 °C. Suspension hematopoietic cells and disaggregated EBs were analyzed by flow cytometry and RNA was extracted or cells were cryopreserved in 10% DMSO/CJ2 medium^54^ before transplantation. For some transplantation experiments (Supplementary Tables 1, 11, 12, 14 and 15), anti-CD34 antibody-conjugated magnetic beads (Miltenyi Biotec) were used according to the manufacturer’s instructions to enrich CD34^+^ cells from disaggregated EBs and deplete cultures of stromal cells before cryopreservation.

### Determination of retinoid treatments for generating iHSCs: screening protocol 2 and mouse cohorts 2 and 3

On day 0, mesoderm was patterned using 4 µM CHIR99021, 3 ng ml^−1^ rh BMP4, 5 ng ml^−1^ rh Activin A, 20 ng ml^−1^ rh FGF2 and 1 µM Thiazovivin or 4 µM CHIR99021 with 30 ng ml^−1^ rh Activin A, 20 ng ml^−1^ rh FGF2 and 1 µM Thiazovivin. From day 1 onward, the medium was changed every 2 days. *HOXA* expression was induced on day 1 as previously described in protocol 1 (3 µM CHIR99021, 4 µM SB431542, 25 ng ml^−1^ rh VEGF, 25 ng ml^−1^ rh SCF and 20 ng ml^−1^ rh FGF2). On day 3, the medium was supplemented with 20 ng ml^−1^ rh BMP4, 50 ng ml^−1^ rh VEGF, 20 ng ml^−1^ rh FGF2, 50 ng ml^−1^ rh SCF, 10 ng ml^−1^ rh IGF2 and 2 µM RETA. The retinoid was removed on the day 5 medium change (control) or RETA supplementation was repeated at 2-day intervals along with fresh medium during the differentiation, as shown in Fig. 4a, at concentrations between 100 nM and 2 µM. From day 5 onward, the medium was supplemented with 2 ng ml^−1^ rh BMP4, 50 ng ml^−1^ rh VEGF, 20 ng ml^−1^ rh FGF2, 50 ng ml^−1^ rh SCF and 10 ng ml^−1^ rh IGF2 with or without RETA. From day 11 of differentiation onward, growth factors included 50 ng ml^−1^ rh VEGF, 50 ng ml^−1^ rh SCF, 50 ng ml^−1^ rh TPO (PeproTech), 10 ng ml^−1^ rh FGF2 and 20 nM SR1, with and without RETA. From days 14 to 16, suspension hematopoietic cells were pooled in some experiments with MACS-enriched CD34^+^ cells from disaggregated EBs, analyzed by flow cytometry and cryopreserved for transplantation.

### Development of a protocol for the generation of hematopoietic cells containing iHSCs from multiple iPS cell lines: protocol 3 and mouse cohorts 4–7

Mesoderm was induced with 4 µM CHIR99021, 30 ng ml^−1^ Activin A, 20 ng ml^−1^ rh FGF2 and 1 µM Thiazovivin and patterned to *HOXA* expression (days 1–3) with 3 µM CHIR99021, 4 µM SB431542, 25 ng ml^−1^ rh VEGF, 20 ng ml^−1^ rh FGF2 and 50 nM RETA. On day 3, the medium was supplemented with 20 ng ml^−1^ rh BMP4, 2 µM RETA, 150 ng ml^−1^ rh VEGF, 20 ng ml^−1^ rh FGF2, 10 ng ml^−1^ rh IGF2 and 10 ng ml^−1^ rh IGF1 (PeproTech). On day 5, rh BMP4 and RETA were reduced to 2 ng ml^−1^ and 100 nM, respectively, and all other cytokines were as on day 3. On day 7, rh VEGF was removed, whilst rh BMP4 and RETA were retained at 2 ng ml^−1^ and 100 nM, respectively, and rh FGF2, rh IGF1 and rh IGF2 were supplemented at 10 ng ml^−1^ each. On day 9, rh SCF was included at 10 ng ml^−1^ and all other cytokines were as on day 7. From day 11 onward, rh BMP was removed and medium changes continued every 2 days. EBs were cultured in 10 ng ml^−1^ each of rh SCF, rh TPO (PeproTech), rh FGF2, rh IGF1, rh IGF2 and 100 nM RETA and 20 nM SR1 (Selleck Chem). From days 14 to 16, suspension hematopoietic cells were analyzed by flow cytometry before being cryopreserved for transplantation.

### Cryopreservation

Cells were cryopreserved in 10% DMSO/CJ2 medium^54^ before transplantation. CJ2 is a protein-free choline chloride-based medium developed for the cryopreservation of mouse oocytes. Cells were frozen using a benchtop controlled-rate freezer, Grant Asymptote EF600 (Grant Technologies), and stored in liquid nitrogen.

### Flow cytometry

Suspension hematopoietic cells and disaggregated EBs were analyzed by flow cytometry. Suspension hematopoietic cells were analyzed separately from cells dissociated from the EBs. EBs were disaggregated by 45-min incubation with 2 mg ml^−1^ collagenase type I (Worthington) at 37 °C followed by mechanical dissociation by passing through a 21-gauge needle attached to a 3-ml syringe. For analysis of mouse tissues, hematopoietic cells were flushed from the bone marrow, spleen and thymus using a 25-gauge needle attached to a 3-ml syringe with PBS to generate single-cell suspensions. Red cell lysis of peripheral blood samples was performed by incubating 100 µl of blood with 10 ml of ammonium chloride lysis buffer (155 mM NH_4_Cl, 12 mM NaHCO_3_ and 0.1 mM EDTA) at 37 °C for 15 min. Cells were pelleted and washed with PBS. For analysis, all samples were resuspended in PBS supplemented with 2% fetal calf serum (FCS). Directly conjugated antibodies directed against cell-surface antigens, detailed in Supplementary Table 24, were used to identify dissociated cells by flow cytometry during differentiation and in single-cell suspensions from hematopoietic tissues and peripheral blood samples from transplanted mice. Samples were incubated with the indicated dilution of antibodies in a volume of 25 µl of PBS supplemented with 2% FCS for 15 min at 4 °C, washed twice with 2 ml of PBS supplemented with 2% FCS and resuspended in 300 µl of PBS supplemented with 2% FCS and 1 µg ml^−1^ propidium iodide to detect dead cells. Flow cytometric analysis used a four-laser BD LSR Fortessa analyzer (Becton Dickinson). The panel of negative controls for flow cytometry is shown in Supplementary Fig. 1. FlowLogic 8 (Inivai Technologies) was used to analyze data and prepare figures.

### CB cells

Samples of human umbilical CB from healthy subjects were obtained from the BDMI National CB Bank, Royal Children’s Hospital, under the auspices of the Royal Children’s Hospital Human Research Ethics Committee (reference 34170A, ID 42470). Mononuclear cells were isolated and cryopreserved for use in transplantation assays.

### Mice

NBSGW mice^24^ were sourced from JAX Mice and Services (stock number 0266220) at The Jackson Laboratory and a colony was established at the Murdoch Children’s Research Institute.

### Transplantation experiments

Differentiated CD34^+^ suspension hematopoietic cells, CD34-enriched swirling EBs or a combination of both were harvested and cryopreserved before transplantation. Cells in most experiments (>85%) were differentiated for 14–16 days before harvesting (Supplementary Tables 1, 11, 12 and 14–17). Cells were thawed and male and female mice aged between 8 and 13 weeks were transplanted by intravenous injection into the tail vein with 5 × 10^5^–2 × 10^6^ cells. The viability of the iPS cell-derived cells was routinely >80%. Samples with viability below 70% were not transplanted. Cryopreserved CB mononuclear cells from four independent cords (0.7–2.7% CD34^+^) were thawed and mononuclear cells estimated to contain 3.5 × 10^2^–2.7 × 10^4^ CD34^+^ cells were transplanted in a similar manner (Supplementary Table 21). Tissues were isolated for analysis from most recipients from 16–24 weeks after engraftment (Supplementary Tables 2 and 3). Single-cell suspensions were generated from peripheral blood, bone marrow (femurs and tibiae), spleen and, where visible, thymic tissue. Cells were analyzed by flow cytometry for surface antigens indicative of erythroid, myeloid, B, T and stem cell compartments. The antibodies used for each lineage are indicated in the legend of Supplementary Table 13. Residual bone marrow and spleen samples from repopulated mice were cryopreserved for further analyses including secondary transplantation.

Bone marrow samples from selected MLE mice were transplanted (3 × 10^5^–2 × 10^6^ total bone marrow cells per mouse) into secondary NBSGW recipients by tail-vein injection and the bone marrow and spleen were analyzed after 13–20 weeks (Supplementary Table 22).

### Transcriptional profiling using scRNA seq

scRNA seq was performed after 14 days of differentiation on a total of 28 samples from RM TOM and PB1.1 BFP samples as outlined in Fig. 3a. Data from suspension hematopoietic cells and EB cells were collected separately. EBs were disaggregated by a 45-min incubation with collagenase type I (Worthington) at 37 °C. Single-cell suspensions were prepared at 1 × 10^6^ cells per ml with at least 90% cell viability and processed by The Victorian Clinical Genetics Service, which prepared the libraries following the 10X Genomics Cell Preparation Guide (www.10xgenomics.com). Sequencing of scRNA was performed using an Illumina Novaseq-6000, aiming for ~300 million reads per sample comprising 6,000–10,000 cells with ~50,000 reads per cell. Selected data from samples from the RM TOM line that were not treated with RETA were published previously^13^.

The FASTQ files generated from the Illumina sequencing were mapped against the human reference genome GRCh38−1.20 using the 10X Cellranger software version 6.0.2 with the Cellranger ‘count’ function. Data from the two iPS cell lines were aggregated with the Cellranger ‘aggr’ function allowing for convenient visualization of genes expressed using the Loupe browser (10X genomics). Other output files generated that were used for bioinformatic analysis consisted of the matrices, barcode and features files found in the ‘filtered_gene_bc_matrices’ folder. Both the Loupe browser and the mapped unprocessed files are accessible from GitHub (https://github.com/jackyyishengli/Ng-2023/).

Visualizations from Fig. 3, Extended Data Figs. 2–4 and Supplementary Figs. 2–3 were generated on the R platform. Seurat version 4.1.2 was used for preprocessing quality control and downstream analysis. Analysis was completed following the Seurat vignette with quality control metrics applied to the raw data. Cells that expressed more than 8 × 10^3^ or fewer than 2 × 10^2^ genes and more than 5 × 10^4^ or fewer than 1 × 10^3^ counts, along with cells that expressed more than 20% mitochondrial, 40% ribosomal and 1.5% mitoribosomal genes, were excluded. Following quality control, the standard Seurat downstream processes were carried out with normalization using log normalization with a scale factor of 10,000 first, followed by identification of the most variable genes of each sample. Integration through the ‘FindAnchors’ and ‘IntegrateData’ functions was performed across all samples to minimize the batch effects seen throughout the 28 PS cell samples. ‘SelectIntegrationFeatures’ was used to determine a list of 2 × 10^3^ genes used in the integration matrix. Scaling was completed next using the ‘ScaleData’ function. Following scaling, the number of dimensions was reduced with principal component analysis and clustering was completed with the ‘FindClusters’ function using the Louvain clustering algorithm. In total, 252,607 cells, comprising 12 RM TOM and 16 PB1.1 BFP samples, passed quality controls.

To identify each cluster within the integrated 28 samples, the ‘FindAllMarkers’ function was used to generate a list of cluster-specific genes for each cluster. These genes were then compared with known markers of a cell type to assign cluster identities (Supplementary Table 4). Differential gene expression analysis between clusters was completed using the ‘FindMarkers’ function, whilst differential genes expressed between samples used a pseudobulk method based on average counts. Here, each single cell within a preselected cell cluster acted as a replicate, thus allowing for the RNA expression level across the cluster to be treated as a bulk RNA sample. Differing conditions of the same cluster could, therefore, use the same analysis strategies as used in bulk RNA seq. To identify the effects of retinoid supplementation, the Voom limma^55,56^ method on the Degust web portal^57^ was used to identify differentially expressed genes in the arterial, hemogenic and HSPC1 clusters identified in Supplementary Fig. 3. Cells from these three clusters were also pooled and reclustered with a higher resolution to investigate the endothelial-to-hematopoietic transition.

ACTINN version 2 (ref. ^27^) was used as an unsupervised neural network based method to identify subsets of the hematopoietically differentiated iPS cell population on the basis of a comparison to a reference dataset of human embryonic-derived and CB-derived endothelial and hematopoietic cell populations (data were taken from a previous study^13^). The ACTINN datasets used in Fig. 3h comprised 27 samples of hematovascular cells from gestational day 22–24 (CS10–11) embryo and YS, day 29–36 (CS14–15) AGM, YS, embryonic liver and placenta, week 6, 8, 11 and 15 embryonic and fetal liver HSPCs and CB HSCs and progenitor cells. The expression matrix for the reference training data and the cell type annotation of the cells are accessible from GitHub (https://github.com/mikkolalab/Human-HSC-Ontogeny). Cells expressing *HLF* and *SPINK2* from the differentiated iPS cells were subsetted and the counts matrix matched to 19 of the 27 reference samples. Results from these 19 datasets are shown in Fig. 3h and Supplementary Table 10.

### Images

Confocal images were captured using a Zeiss LSM 900 laser scanning confocal microscope with Zeiss Blue software (Zeiss, version 2.1). Images for figures were assembled in Adobe Illustrator 2020 (version 24.1). Adjustments to brightness and contrast were the only image manipulations performed. The diagrams in Figs. 1a, 2a,b, 3a and 4a and Extended Data Figs. 1a and 7a were created in part using BioRender.com.

### Statistical analysis

Experiments were analyzed using GraphPad Prism versions 7–10 and Microsoft Excel. The mean and s.e.m. are shown with the number of independent replicates in each case in the figure legend, on the figure or in the text. Tests for statistical significance are listed in the figure legend of each experiment. One-way analysis of variance (ANOVA)-based statistics (Kruskal–Wallis for nonparametric distributions) were used for experiments with multiple comparisons of one or more grouped variables, accompanied by post hoc tests indicated as appropriate by the software (Dunn’s). A two-tailed Fisher’s exact test was used to compare groups in contingency tables. Mann–Whitney two-tailed tests were used to compare unpaired nonparametric groups and two-tailed Wilcoxon signed rank tests were used for paired nonparametric groups. The reproducibility of the data is captured by the number of experimental replicates, as listed in figure legends. No statistical method was used to predetermine sample size.

### Reporting summary

Further information on research design is available in the Nature Portfolio Reporting Summary linked to this article.

## Online content

Any methods, additional references, Nature Portfolio reporting summaries, source data, extended data, supplementary information, acknowledgements, peer review information; details of author contributions and competing interests; and statements of data and code availability are available at 10.1038/s41587-024-02360-7.

## Supplementary information

Supplementary Information Supplementary Figs. 1–5, Results 1–4 and Discussion.

Reporting Summary

Supplementary Table 1 Engraftment data from mice transplanted in cohort 1. Summary data are included on the second worksheet.

Supplementary Table 2 Time of analysis after transplantation of mice by experimental cohort.

Supplementary Table 3 Correlation of level of human engraftment in BM of MLE animals, time of analysis and gender of recipient.

Supplementary Table 4 Cluster-specific gene expression in integrated sample data from scRNA seq analysis shown in Fig. 3c.

Supplementary Table 5 Cell composition of the endothelial to hematopoietic transition clusters shown in Fig. 3f,g, Extended Data Figs. 2a–c, 3 and 4, and Supplementary Fig. 3a.

Supplementary Table 6 Genes upregulated or downregulated in response to RETA shown in the Venn diagrams in Supplementary Fig. 3d,e are listed.

Supplementary Table 7 Differentially expressed genes between retinoid-treated and nontreated arterial cluster cells (Supplementary Fig. 3).

Supplementary Table 8 Differentially expressed genes between retinoid-treated and nontreated hemogenic endothelium cluster cells (Supplementary Fig. 3).

Supplementary Table 9 Differentially expressed genes between retinoid-treated and nontreated HSPC1 cells (Supplementary Fig. 3).

Supplementary Table 10 Number and percentage of *HLF*^+^*SPINK2*^+^ iPS cell-derived cells with the closest transcriptional similarity to each human embryo and CB reference dataset as determined by ACTINN machine learning (Fig. 3h).

Supplementary Table 11 Engraftment data from mice transplanted in cohort 2. Summary data are included on the second worksheet.

Supplementary Table 12 Engraftment data from mice transplanted in cohort 3. Summary data are included on the second worksheet.

Supplementary Table 13 Phenotypic characteristics of MLE mice from cohort 1–3 transplantation experiments.

Supplementary Table 14 Engraftment data from mice transplanted in cohort 4. Cells were differentiated using protocol 3. Summary data are included on the second worksheet.

Supplementary Table 15 Engraftment data from mice transplanted in cohort 5. Cells were differentiated using protocol 3. Summary data are included on the second worksheet.

Supplementary Table 16 Engraftment data from mice transplanted in cohort 6. Cells were differentiated using protocol 3. Summary data are included on the second worksheet.

Supplementary Table 17 Engraftment data from mice transplanted in cohort 7.

Supplementary Table 18 Engraftment data comparing MLE frequency in mice transplanted with SN blood cells or in those receiving cells that also included MACS-enriched CD34^+^ cells from the EBs.

Supplementary Table 19 Phenotypic characteristics of MLE mice from cohort 4–7 transplantation experiments. Cells were differentiated using protocol 3.

Supplementary Table 20 Summary of thymic-engrafted and/or mediastinal LN-engrafted mice. Subsets of T cells represent percentages of human cells detected in the thymus and/or mediastinal LN.

Supplementary Table 21 Phenotypic characteristics of CB-engrafted mice.

Supplementary Table 22 Outcome of secondary engraftment experiments using iHSC-engrafted and CB-engrafted primary recipient BM.

Supplementary Table 23 Composition of SPELS medium.

Supplementary Table 24 Antibodies used for flow cytometry.

Supplementary Data 1 Statistical source data for supplementary figures.

## Source data

Source Data Figs. 2 and 4–6 and Extended Data Figs. 1 and 5–10 Statistical source data.

## Data Availability

RNA seq data supporting this study were deposited to the Gene Expression Omnibus (GEO) under accession code GSE232710. Published datasets of human embryonic tissues used in this study are available from the GEO under accession codes GSE162950 and GSE135202. The reference datasets used for the ACTINN analysis are available from figshare (https://figshare.com/articles/ACTINN/8967116)^58^. Seurat data objects and codes are available from GitHub (https://github.com/jackyyishengli/Ng-2023/). Source data are provided with this paper.
