# Supplementary material for: Long-term engrafting multilineage hematopoietic cells differentiated from human induced pluripotent stem cells
Source: Nat Biotechnol. 2024 Sep 2;43(8):1274–87. doi: 10.1038/s41587-024-02360-7 (PMC12339382; doi:10.1038/s41587-024-02360-7)
Supplement: Supplementary file 1 — Supplementary Figs. 1–5, Results 1–4 and Discussion. [file 41587_2024_2360_MOESM1_ESM.pdf]

nature biotechnology

Supplementary information https://doi.org/10.1038/s41587-024-02360-7

Long-term engrafting multilineage hematopoietic cells differentiated from human induced pluripotent stem cells

In the format provided by the authors and unedited

Supplementary Figures and Legends

SUPPLEMENTARY FIGURE 1

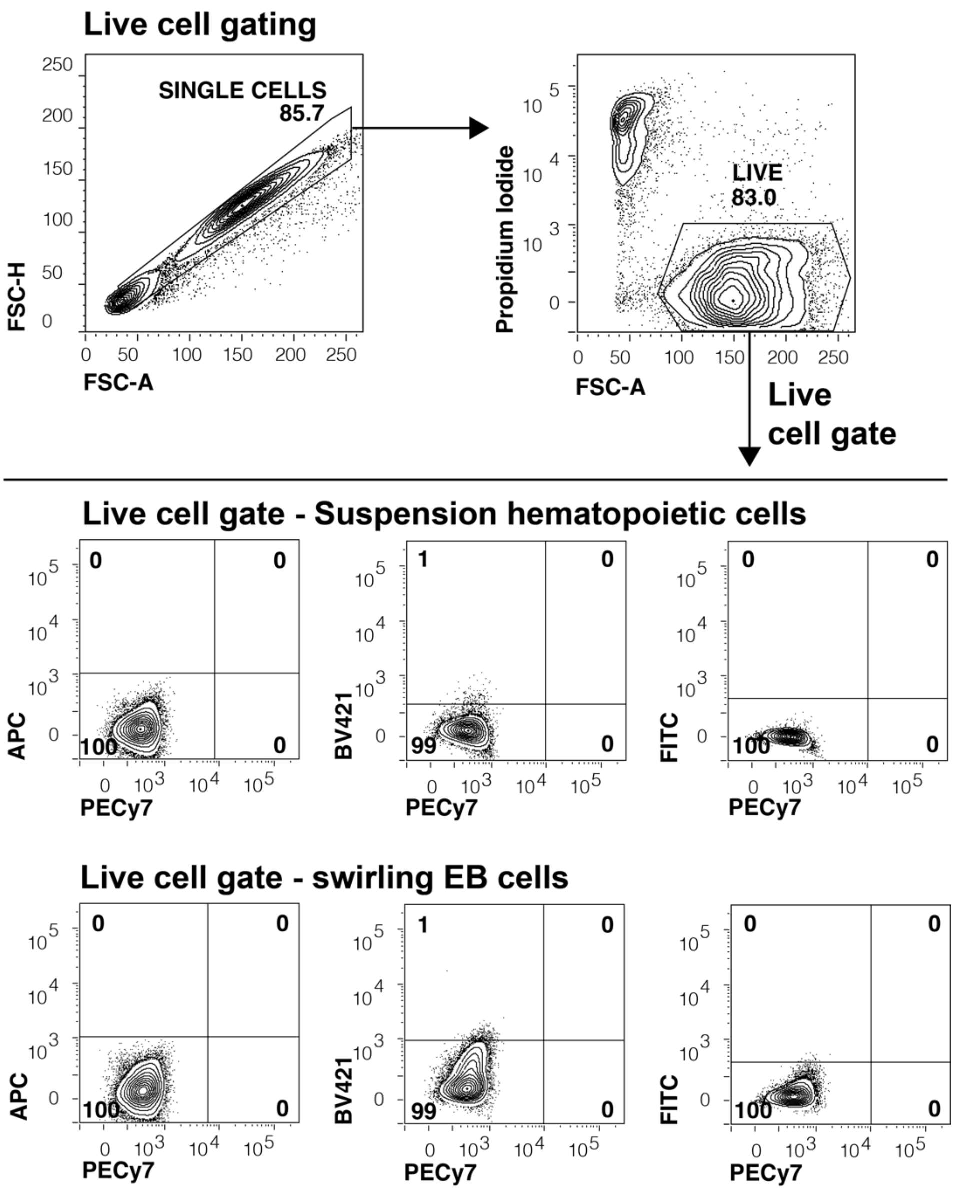

Supplementary Figure 1. Flow cytometry live cell gating strategy and negative control samples. Data collected in the indicated fluorochrome channels for unstained suspension hematopoietic cells and disaggregated swirling embryoid body (EB) cells. See also Fig. 1d - e. The same strategy was used for evaluation of iPSC engraftment in mouse hematopoietic tissues shown in Figs. 4d– g, 5i - j, 6c and Extended Data Figs. 5b - c and 8a - d.

SUPPLEMENTARY FIGURE 2

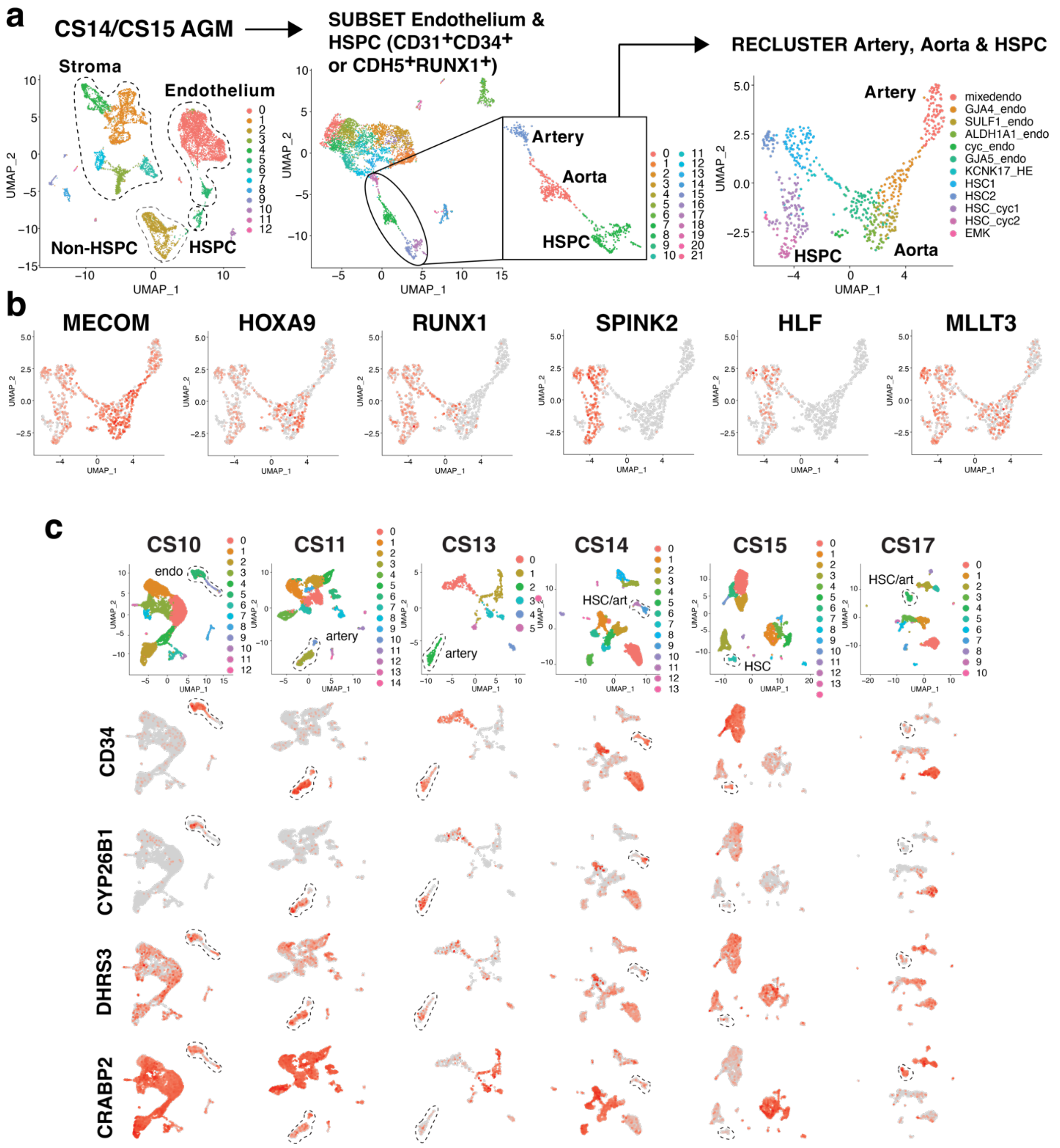

Supplementary Figure 2. Transcriptional profiling of human embryo arterial and hematopoietic stem/progenitor cell (HSPC) populations and expression of retinoid dependent genes in CS10 – CS17 human embryos. (**a**) Left panel depicts UMAP of 11,877 single cells from CS14 and CS15 embryos [13], with endothelium, stroma, HSPC and non-HSPC populations indicated. Middle panel shows subset analysis of 4,532 endothelial cells and HSPCs, selected on expression of either CD34 and CD31 or RUNX1 and CDH5. In the right panel, 634 cells from artery, aorta and HSPCs were reclustered. (**b**) Feature plots of the reclustered artery, aorta and HSPCs showing expression of human embryonic HSC signature genes [13]. (**c**) Feature plots of *CD34* and selected retinoid responsive genes throughout human embryogenesis from CS10 – CS17. Key endothelial, arterial and HSC containing populations are circled with dashed lines. Note overlap of these cell clusters expressing *CD34*, marking endothelium and HSPCs, with retinoid responsive genes.

SUPPLEMENTARY FIGURE 3

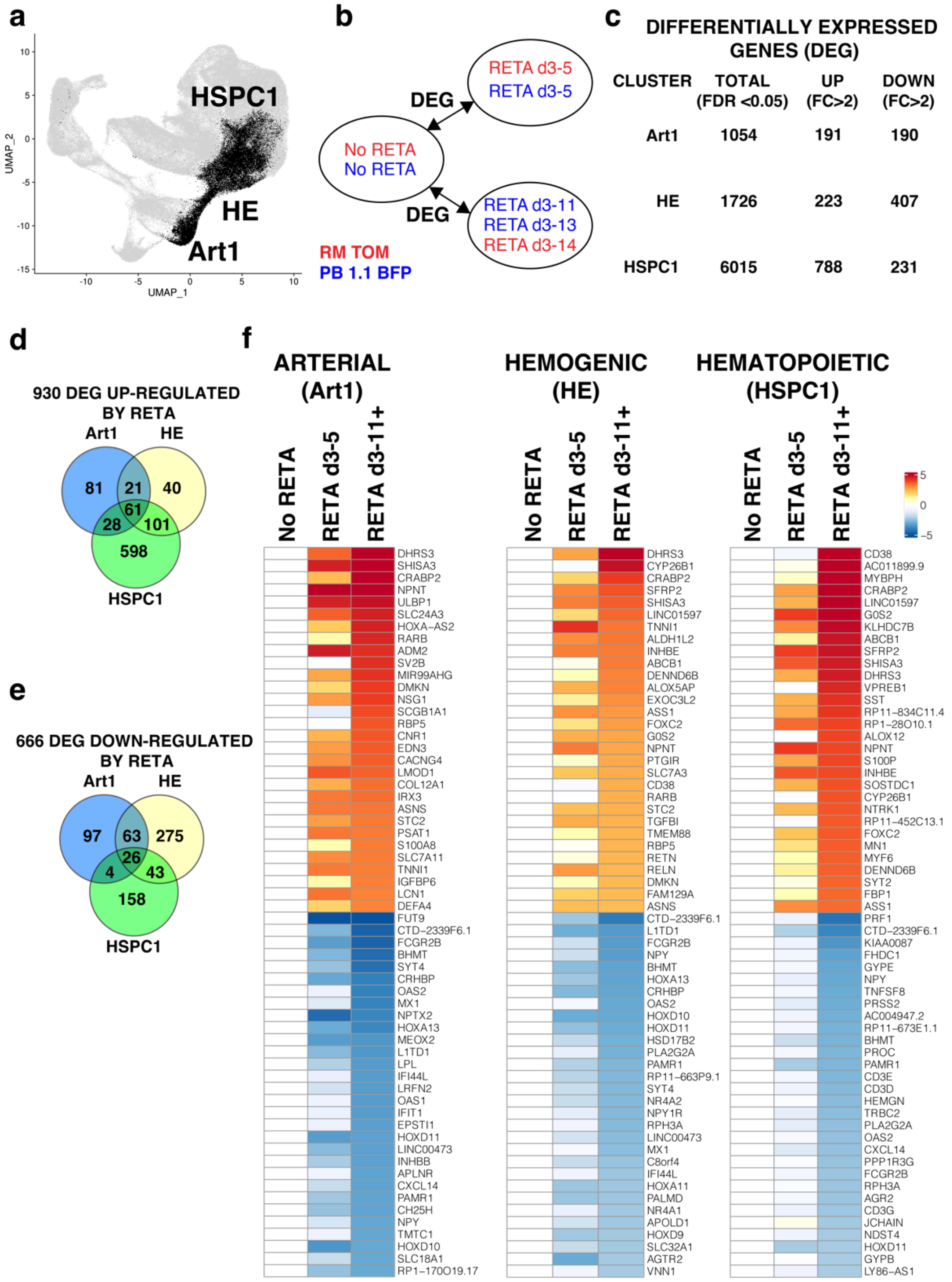

Supplementary Figure 3. Differentially expressed genes in cultures exposed to different durations of retinyl acetate (RETA). (**a**) UMAP showing arterial (Art1), hemogenic (HE) and stem/progenitor clusters (HSPC1) differentiated from RM TOM and PB1.1 BFP iPSC lines. Cell numbers and composition are provided in Supplementary Table 5. Integrated data from all samples was used. Differential gene expression was determined by comparing (**b**) genes expressed following a pulse of RETA from day 3 – 5 or for a prolonged period (day 3 – day 11, day 13 and day 14) with cells not treated with RETA. (**c**) Number of differentially expressed genes in each cluster with a false discovery rate (FDR) <0.05 and the subset of these genes up- or down-regulated by a fold change (FC) > 2 are indicated. (**d**, **e**) Venn diagrams demonstrating the overlap in genes (**d**) up- and (**e**) down-regulated in response to retinoid exposure in each cluster. (**f**) Heatmaps displaying the 30 most highly up- and down-regulated genes in each cluster in response to RETA compared to No RETA. Color bar indicates log fold-change compared to 'No RETA'. See also Supplementary Tables 6 – 9 for gene lists.

SUPPLEMENTARY FIGURE 4

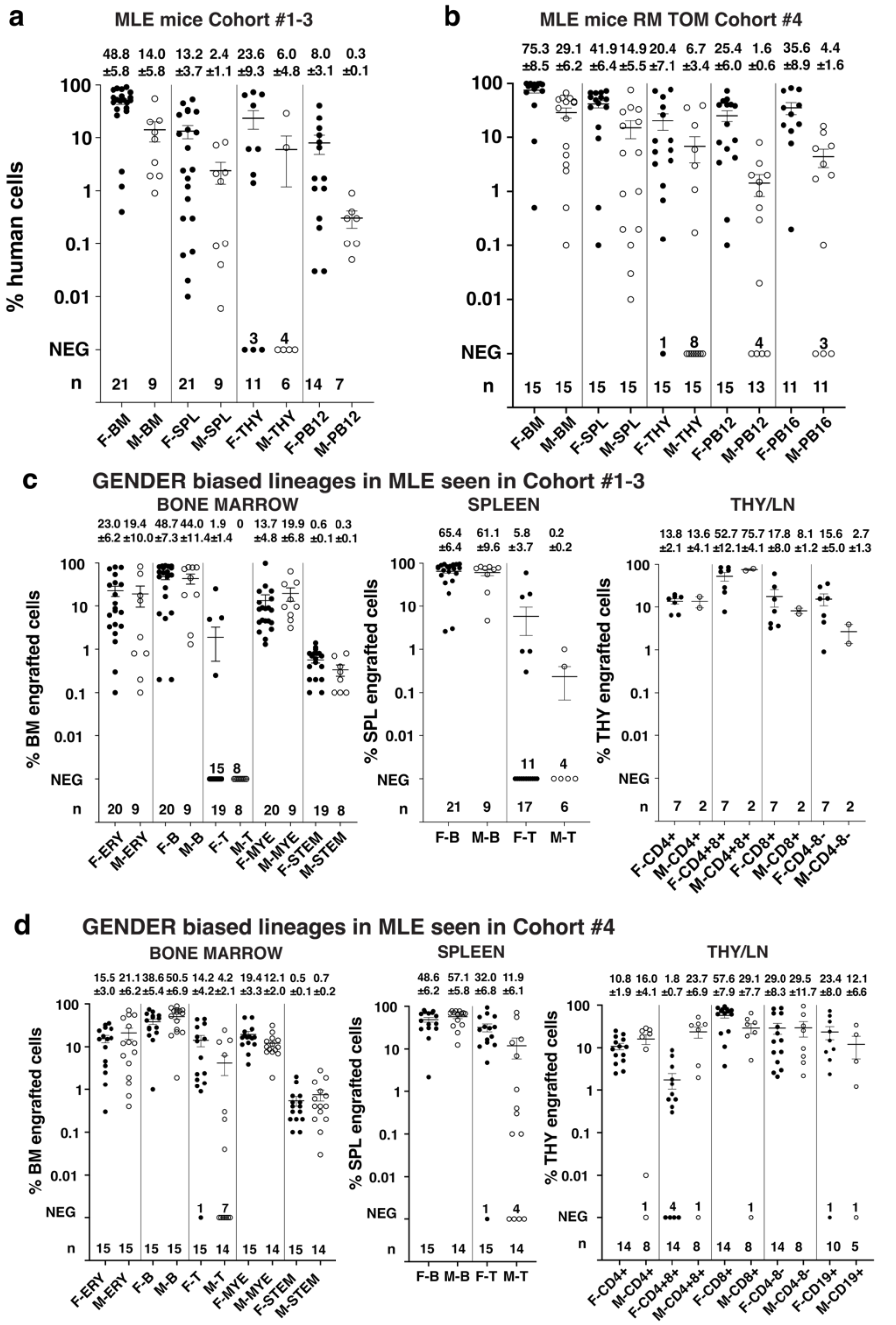

Supplementary Figure 4. Gender differences in MLE mice. (**a**, **b**) Tissue distribution in (**a**) cohort #1-3 and (**b**) cohort #4 multilineage engrafted female and male mice. All mice except one from cohort #1 – 3 were engrafted with RM TOM cells. Female vs male cohort #1-3 mice, bone marrow engraftment, P=0.0041; peripheral blood 12 week engraftment, P=0.0357. Female vs male cohort #4 mice, bone marrow engraftment, P=0.0003; spleen, P=0.0044; thymus, P=0.0133; peripheral blood 12 week engraftment, P<0.0001; peripheral blood 16 week engraftment, P=0.0004. Comparing cohort #1-3 with cohort #4 female mice, engraftment was greater in cohort #4 female mice in the bone marrow, P=0.0030; spleen, P=0.0006; and peripheral blood at 12 weeks, P=0.0266. Engraftment comparisons between male mice were not statistically significant. Mann-Whitney t-tests. (**c**, **d**) Distribution of lineages in bone marrow, spleen, and thymus/lymph node in (c) cohort #1-3 and (d) cohort #4 multilineage engrafted female and male mice. (**c**) In cohort #1-3 mice, the proportions of erythroid, myeloid, B cell and stem cells were similar in male and female recipients. (**d**) In cohort #4 mice, T cell engraftment was greater in female mice in the bone marrow, P=0.0049; spleen, P=0.0065; and thymic $CD4^+CD8^+$ cells were more abundant in male mice; P= 0.0058. Myeloid cells were more abundant in the BM of female mice, P=0.0378. Thymic $CD8^+$ cells were more abundant in female mice, P=0.0352. Mann-Whitney t-tests.

SUPPLEMENTARY FIGURE 5

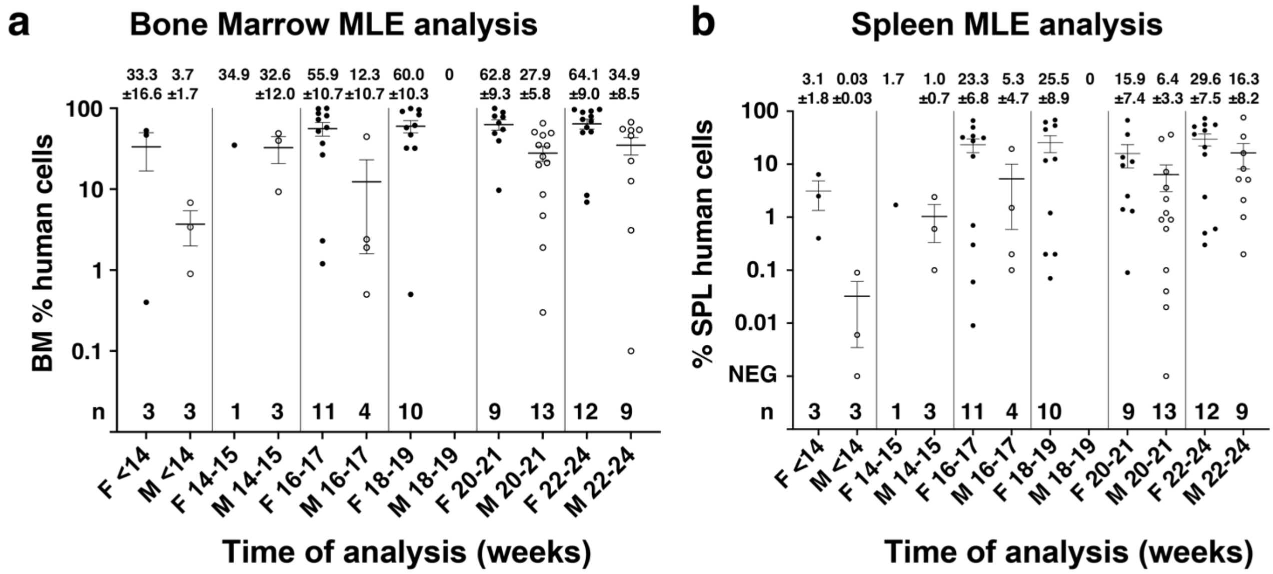

Supplementary Figure 5. Correlation between time of tissue analysis post-transplantation and human cell engraftment in (**a**) bone marrow (n = 78) and (**b**) spleen (n = 78) in multilineage engrafted recipients of iHSCs, stratified for sex of the recipient. Panels show a trend of increasing human cell contribution in recipients engrafted for longer periods. The plateau appears earlier for female (18-19 weeks) than for male (at least 22-24 weeks) recipients. For mice analyzed after 20 weeks, the level of engraftment was higher for female mice in the bone marrow (*P*=0.0002) and the spleen (*P*=0.0374). Mann-Whitney t-tests.

Supplementary Results 1

Differentiation of iPSCs to CD34 expressing hematopoietic cells

Induced pluripotent stem cells were differentiated to hematopoietic cells using a swirling embryoid body (EB) protocol, in which iPSCs were seeded into dishes that were incubated on a rotating platform [13, 22] (see Methods and Extended Data Fig. 1a for protocol details, Fig. 1a, b). Mesoderm was induced in albumin-free SPELS medium (made in house, see Methods) using a combination of the WNT-agonist CHIR99021 (CHIR), FGF2, BMP4 and/or ACTIVIN A for 24 hours (Fig. 1a). Subsequent patterning to induce the expression of *HOXA* genes was achieved by culture for 48 hours with CHIR and the ALK-kinase inhibitor SB431542 (SB) [18]. From day 3, mesoderm was further differentiated to hemogenic endothelium, with or without a 2-day pulse of a retinoic acid precursor, retinyl acetate (RETA) or retinol (ROL). From day 7, cells undergoing an endothelial to hematopoietic transition were visible as protrusions on the surface of the embryoid bodies, reminiscent of intra-arterial hematopoietic clusters of blood cells that are seen emerging from the aorta in the embryonic AGM [10, 11] (Fig. 1c). From day 9, these cellular accumulations broke away from the embryoid bodies, shedding blood cells into the medium from day 11 (Fig. 1c). Cultures at day 14 comprised a dominant blood cell suspension with most cells expressing CD34, CD90, CD44 and KIT (Fig. 1d and Supplementary Fig. 1). The embryoid body-derived fraction consisted of stroma, endothelium, and hematopoietic cells that resembled those shed into the medium (Fig. 1e and Supplementary Fig. 1). A small proportion of the hematopoietic cells expressed CXCR4 or CD73, reflecting their recent emergence from an endothelial precursor (Fig. 1e). The endothelial populations present at this time included $CXCR4^+CD73^+$ arterial cells, $CXCR4^-CD73^{hi}$ venous cells and $CXCR4^-CD73^-$ hemogenic cells [15]. From day 14 – 16, the suspension hematopoietic cells were cryopreserved for further analyses (Fig. 1d). In some experiments, $CD34^+$ cells enriched from embryoid bodies by magnetic bead separation (Fig. 1e) were also cryopreserved.

Typically, 2.0 x 10^6 iPSCs were differentiated per 60 mm dish to give an approximate total of 1.4 x 10^7 $CD34^+$ hematopoietic cells per dish in suspension by day 14, a yield of 7 $CD34^+$ cells (7.1 ± 0.8, n=3) for each input iPSC. When performed, magnetic bead separation yielded approximately 1.0 x 10^6 $CD34^+$ cells per 60 mm dish, and these cells were usually pooled with the hematopoietic cells harvested from the suspension phase of the culture prior to cryopreservation.

Supplementary Results 2

Multilineage engrafting cells require retinoids during iPSC differentiation

Groups of mice (totaling 134, denoted cohort #1) were injected with RM TOM cells differentiated under one of 12 mesoderm induction and patterning protocols (Extended Data Fig. 3a, Supplementary Table 1). Human cell contributions in the bone marrow and spleen were determined post

transplantation based on coincident expression of the TOMATO reporter with human lympho-myeloid (CD45 and/or CD43) and erythroid (GYPA) cell surface markers by flow cytometry (Supplementary Table 1). For cohort #1, most animals were analyzed >12 weeks after transplantation (85%) and half were >16 weeks post transplantation (49%). Analysis time for mice in all cohorts is shown in Supplementary Table 2. Across the 12 conditions, 103/134 (76.9%) of animals had detectable human cells in the bone marrow with 45/103 (43.7%) also having human cells in the spleen (Extended Data Fig. 1c and Supplementary Table 1). Flow cytometry revealed different patterns of lineages in individual mice, suggesting that there was a hierarchy of engrafting cells. Most frequent were myeloid restricted stem cells that gave low-level bone marrow engraftment (human cells, 0.7±0.1%) in 58/134 transplant recipients, or myelo-lymphoid stem cells that led to mixture of bone marrow myeloid cells (human cells, 0.5±0.1%) accompanied by B lymphocytes in the spleen (human cells, 0.02±0.003%) in 30/134 mice (Extended Data Fig. 1d). A small number of mice (3/134) were engrafted predominantly by a higher proportion of B lymphoid cells (8.7±4.4% human cells in bone marrow, and 0.4±0.3% in spleen) (Extended Data Fig. 1d). However, 12 mice (12/134) displayed features of engraftment by a more primitive, more highly proliferative stem cells, displaying multilineage engraftment (MLE) with erythroid, myeloid and lymphoid reconstitution with over 80% human cells occupying the bone marrow in some cases (average of 38.0±8.9% human cells in bone marrow, and 9.0±4.1% in spleen) (Extended Data Fig. 1d).

Supplementary Results 3

Transcriptional effects of retinoid treatment on *in vitro* differentiated iPSCs

The addition of retinoids affected gene expression in all three clusters, with 930 genes up regulated and 666 genes down-regulated > 2-fold by RETA (Supplementary Fig. 3a – f and Supplementary Tables 6 – 9). Up-regulated genes in the ART1 cluster included those associated with retinoic acid metabolism such as *CYP26B1*, *DHRS3*, *CRABP2*, *RARB* and *RARG*, modulators of WNT and FGF signalling such as *SHISA3*, *DKK1*, *RSPO1* and *WNT4*, as well as genes associated with vascular and hematopoietic development such as *FOXC2* and *CD38* (Supplementary Fig. 3f and Supplementary Table 7). Retinoid metabolism genes were similarly up-regulated in the HE cluster, as were genes related to Tube Development (gene ontology term GO:0035295, FDR 3.8 x 10^{-6}) (Supplementary Fig. 3f and Supplementary Table 8). Genes enriched in the HSPC1 cluster included those associated with Positive Regulation of Multicellular Organismal Process (gene ontology term GO:0051240, FDR 1.04 x 10^{-16}), and Genes Upregulated in HL-60 Myeloid Cells in Response to Retinoic Acid (FDR 1.03 x 10^{-23}) (Supplementary Fig. 3f and Supplementary Table 9).

Interestingly, expression of many of these genes was only induced if the retinoids were included until at least day 11 of differentiation (Supplementary Fig. 3f and Extended Data Fig. 2d). When we

examined scRNA seq data from human embryos, we observed the expression of a similar cohort of retinoic acid responsive genes from gestational day 22 – 40 (CS10 – 17) (Supplementary Fig. 2c). We interpreted this to imply that the developing embryo is exposed to retinoid signaling for a prolonged period during gestation, rather than just for the limited time (from day 3 – day 5) that we had assessed in cohort #1 transplanted mice.

We also looked to see whether expression of specific genes was down-regulated following exposure to retinoids. In the Art1 cluster, genes related to Defense Response to Virus (GO:005160, FDR 5.01 x 10^{-18}), that predominantly include interferon signaling genes were reduced (Supplementary Table 7). We also observed a reduced expression of posterior *HOX* cluster genes, contrasting with the documented effects of retinoic acid to increase the expression of anterior *HOX* cluster genes [49].

Supplementary Results 4

Transcriptional profiles of iPSC-derived hematopoietic cells resemble those of hematopoietic cells from the AGM

To determine the similarity between the iPSC-derived hematopoietic stem cell like populations and similar populations found in the human embryo, we compared the transcriptomes of iPSC-derived cells from the HSPC1-3 clusters that co-expressed *HLF* and *SPINK2*, with those of *HLF*$^{+}$*SPINK2*$^{+}$ cells from CS14 and CS15 embryos, using the scorecards developed by the Mikkola laboratory as templates[13] (Extended Data Figs. 3 and 4). Examining the 'Nascent HSC' scorecard, we confirmed expression of the six HSC signature genes in the iPSC-derived cells, although the proportion of cells expressing *HOXA9*, *MLLT3* and *MECOM* were a little lower than in the CS14 and CS15 embryo reference samples. Similarly, there were some genes enriched in HSCs or shared with endothelium that were expressed in a higher percentage of cells in embryo samples, such as *STAT5A*, *GATA2*, *SELP*, *ALDH1A1*, *PROCR* and *EMCN* (Extended Data Fig. 3).

Both the 'HSC transcription factor' and 'HSC maturation' scorecards showed high degrees of concordance in gene expression between embryo and iPSC-derived samples (Extended Data Fig. 3). The percentage of *GATA3*, *HOXA7*, *PBX1*, *PVLAP* and *CSF1R* expressing cells was a little less in the iPSC-derived samples.

The expression pattern of genes in the 'HSPC waves' scorecard reflected similarity between the embryo and iPSC-derived samples, but we noted that the percentage of cells expressing definitive transcriptional regulation and definitive HSC genes was lower in the iPSC-derived samples (Extended Data Fig. 3). The 'Hematopoietic cell identity' scorecard confirmed the predominant expression of HSC rather than lineage marking genes in the iPSC-derived cells (Extended Data Fig. 3). Expression of the 'Liver *SPINK2*$^{+}$ genes' were very low in all samples except for *PKIB* (Extended Data Fig. 4). The 'Proliferation and metabolic activity' scorecard showed similar gene expression between cell

sources. The 'Signaling' scorecard showed broad concordance between embryo and iPSC-derived samples with the notable exceptions of the retinoid metabolising enzyme *ALDH1A1*, the BMP-responsive transcription factor *ID3*, and some differences in the balance of JAK-STAT signaling genes (Extended Data Fig. 4). The transcriptional regulators and the hematopoietic lineage genes in the 'Endothelial to hematopoietic transition' scorecards (Extended Data Fig. 4d-f) were concordantly expressed in iPSC-derived and embryo samples. However, iPSC-derived arterial cells (Extended Data Fig. 4f) expressed very low levels of the aortic (pre-hemogenic endothelium) genes.

Some differences were observed between the RM TOM and PB1.1 BFP cell lines, with a higher percentage of cells in the RM TOM differentiated cells expressing genes from the 'HSC enriched' and 'HSC shared with endothelium' sections of the 'Nascent HSC' scorecard (Extended Data Fig. 3). Similarly, increased proportions of RM TOM cells expressed 'HSC maturation' scorecard genes and one of the genes associated with early-stage HSCs, *DDIT4*, a regulator of cell growth and survival, on the 'HSPC waves' scorecard (Extended Data Fig. 3). From the 'Hematopoietic cell identity' scorecard, *CDH5* and *LYZ* were expressed in a higher percentage of RM TOM cells (Extended Data Fig. 3). In the 'Liver *SPINK2*$^{+}$ genes' scorecard, LTB was expressed more highly in PB1.1 BFP derived cells. Examination of the 'Signaling' scorecard showed that downstream NOTCH signaling targets *HES1* and *HES4*, *RARA*, *RXRA* were more prominent in RM TOM cells. Higher expression of the endothelial gene *TJP1* was also seen in RM TOM cells in the 'Endothelial to hematopoietic transition' scorecard. Few genes from the scorecard analyses appeared to be retinoid signaling responsive (the HE gene *KCNK17*, the transcription factor *GATA2*, and the BMP4 target *ID1*), all of which were expressed in a higher percentage of RM TOM cells (Extended Data Fig. 4).

Supplementary Discussion

Secondary engraftment from iHSC and CB transplanted mice

We observed secondary engraftment from 6/12 primary mice engrafted with iHSCs and from 2/5 primary mice engrafted with CB HSCs with similar outcomes observed from primary recipients engrafted with cells generated by different protocols (Discussion and Supplementary Table 22). Engraftment was at a low level and restricted to myeloid lineages, although one iHSC secondary transplant recipient displayed B, T and myeloid lineages in the BM, spleen and thymus. Secondary engraftment was seen from primary mice exhibiting high levels of human cells in the BM (over 50%), but this correlation was not absolute, with some iHSC and CB primary animals engrafted with over 90% human cells that did not give secondary engraftment. Secondary engraftment from iHSCs was seen with primary recipients of RM TOM cells from cohorts #1, #2 and #4 and from a PB1.1 BFP engrafted mouse from cohort #5.

Analysis of CB and AGM engrafted immune deficient mice has revealed a marked disparity in the self-renewal capacity of HSCs from these primary sources [38]. Secondary transplantation of CB engrafted NSG mouse bone marrow following high level primary engraftment with 5 x 10^4 $CD34^+$ cells, required 7.5 x 10^6 primary bone marrow cells to achieve an average peripheral blood engraftment of 2.7% after 5 months in 6 recipients, whilst secondary transplantation of 3.6 x 10^6 primary BM cells only engrafted 2/4 recipients with 0.45% peripheral blood chimerism. Similar results following serial transplantation of CB engrafted BM have been reported by others. Liu and colleagues re-transplanted 3 x 10^4 $CD34^+$ CB cells purified from 70% of the BM of primary NSG recipients, but reported less than 0.5% chimerism after 12 w, in only 20% of recipients [37]. MacIntosh and colleagues found that a large primary engrafting dose of 2.5 x 10^5 $CD34^+$ CB cells in NBSGW mice was required to achieve low levels of peripheral blood chimerism (2.1%) in secondary recipients at 12 w [24]. In another study in which a large number (2.5 x 10^6) of $CD34^+$ CB cells engrafted into NBSGW mice were secondarily transplanted, low levels of myeloid lineage restricted secondary engraftment were seen despite each recipient receiving 3 x 10^7 bone marrow cells [41]. Given that we only transplanted 0.3 - 2 x 10^6 BM cells into secondary recipients, below the numbers required for CB engraftment in the studies referenced, it is not surprising that we observed limited, myeloid-restricted engraftment in recipients of 2/5 primary CB engrafted BM (Supplementary Table 22). However, the Medvinsky laboratory showed that NSG mice engrafted with AGM-derived HSCs reliably achieved secondary engraftment with transplantation of 5 x 10^5 BM cells, although the level of peripheral blood engraftment varied between AGM samples from 5.0 – 48.0% [38].With the caveat that our study and that of Medvinsky used different strains of immune deficient mice (NSG versus NBSGW), transplantation of cells from iHSC engrafted bone marrow might have been expected to result in higher proportions of secondarily engrafted recipients if the iHSCs displayed the same degree

of self-renewal as AGM generated HSCs. Our results suggest that iHSCs generate a bone marrow HSC compartment with similarly functioning stem cells to CB but may lack the ability for significant expansion that marks AGM-derived HSCs.
