# Supplementary material for: Long-term engrafting multilineage hematopoietic cells differentiated from human induced pluripotent stem cells
Source: Nat Biotechnol. 2024 Sep 2;43(8):1274–87. doi: 10.1038/s41587-024-02360-7 (PMC12339382; doi:10.1038/s41587-024-02360-7)
Supplement: Supplementary file 2 — Reporting Summary [file 41587_2024_2360_MOESM2_ESM.pdf]

nature portfolio

Corresponding author(s): Andrew G Elefanty and Elizabeth S Ng

Last updated by author(s): Jul 15, 2024

Reporting Summary

Nature Portfolio wishes to improve the reproducibility of the work that we publish. This form provides structure for consistency and transparency in reporting. For further information on Nature Portfolio policies, see our Editorial Policies and the Editorial Policy Checklist.

Statistics

For all statistical analyses, confirm that the following items are present in the figure legend, table legend, main text, or Methods section.

n/a	Confirmed	
☐	☒	The exact sample size (*n*) for each experimental group/condition, given as a discrete number and unit of measurement
☒	☐	A statement on whether measurements were taken from distinct samples or whether the same sample was measured repeatedly
☐	☒	The statistical test(s) used AND whether they are one- or two-sided<br>*Only common tests should be described solely by name; describe more complex techniques in the Methods section.*
☐	☒	A description of all covariates tested
☐	☒	A description of any assumptions or corrections, such as tests of normality and adjustment for multiple comparisons
☐	☒	A full description of the statistical parameters including central tendency (e.g. means) or other basic estimates (e.g. regression coefficient) AND variation (e.g. standard deviation) or associated estimates of uncertainty (e.g. confidence intervals)
☐	☒	For null hypothesis testing, the test statistic (e.g. *F*, *t*, *r*) with confidence intervals, effect sizes, degrees of freedom and *P* value noted<br>*Give P values as exact values whenever suitable.*
☒	☐	For Bayesian analysis, information on the choice of priors and Markov chain Monte Carlo settings
☒	☐	For hierarchical and complex designs, identification of the appropriate level for tests and full reporting of outcomes
☒	☐	Estimates of effect sizes (e.g. Cohen's *d*, Pearson's *r*), indicating how they were calculated

Our web collection on statistics for biologists contains articles on many of the points above.

Software and code

Policy information about availability of computer code

Data collection	No software used to collect data
Data analysis	Single cell RNA sequencing data analysis described in detail in Methods, Transcriptional profiling using scRNA sequencing. Software used:<br>10X Cellranger software version 6.0.2<br>Under the R platform, Seurat version 4.1.2 was used to analyse single cell RNA sequencing data.<br>ACTINN version 2.

For manuscripts utilizing custom algorithms or software that are central to the research but not yet described in published literature, software must be made available to editors and reviewers. We strongly encourage code deposition in a community repository (e.g. GitHub). See the Nature Portfolio guidelines for submitting code & software for further information.

Data

Policy information about availability of data

All manuscripts must include a data availability statement. This statement should provide the following information, where applicable:

- Accession codes, unique identifiers, or web links for publicly available datasets
- A description of any restrictions on data availability
- For clinical datasets or third party data, please ensure that the statement adheres to our policy

RNA sequencing data supporting this study have been deposited at the Gene Expression Omnibus (GEO) under accession code GSE232710. Published data sets of

human embryonic tissues used in this study are available at the GEO under accession codes GSE162950 and GSE135202. The reference datasets used for the ACTINN analysis are available from https://figshare.com/articles/ACTINN/8967116. Seurat data objects and codes are available at GitHub: https://github.com/jackyyishengli/Ng-2023/.

Human research participants

Policy information about studies involving human research participants and Sex and Gender in Research.

Reporting on sex and gender	No studies involving human research participants
Population characteristics	Not applicable
Recruitment	Not applicable
Ethics oversight	Not applicable

Note that full information on the approval of the study protocol must also be provided in the manuscript.

Field-specific reporting

Please select the one below that is the best fit for your research. If you are not sure, read the appropriate sections before making your selection.

☒ Life sciences ☐ Behavioural & social sciences ☐ Ecological, evolutionary & environmental sciences

For a reference copy of the document with all sections, see nature.com/documents/nr-reporting-summary-flat.pdf

Life sciences study design

All studies must disclose on these points even when the disclosure is negative.

Sample size	The number of animals transplanted were sufficient to allow the identification of differentiation conditions that yielded statistically different engraftment outcomes. No prior sample size calculation was performed.
Data exclusions	Data from no animals was excluded from analysis of transplantation data. For RNA sequencing analyses, cells that expressed more than 8 x 10(3) or fewer than 2 x 10(2) genes, more than 5 x 10(4) or fewer than 1 x 10(3) counts, along with cells that expressed more than 20% mitochondrial, 40% ribosomal and 1.5% mitoribosomal genes were excluded.
Replication	For the transplantation experiments in Cohort #1 , results were the synthesis of 11 experiments using RM TOM cells sourced from 7 independent differentiations, and a total of 134 animals. For Cohort #2 transplantations, 10 experiments were performed using RM TOM cells sourced from 6 independent differentiations, and a total 103 animals. Cohort #3 transplantations involved 8 experiments using PB1.1 BFP cells from 6 independent differentiations, and a total of 79 animals. Transplantation experiments in Cohort #4 - 7 used an improved version of the iPSC differentiation protocol. Cohort #4 transplantations used RM TOM cells from 7 experiments sourced from 6 independent differentiations, and a total of 62 mice. Cohort #5 transplantations used PB1.1 BFP cells from 4 experiments sourced from 5 independent differentiations, and a total of 23 mice. Cohort #6 transplantations used PB5.1 cells from 4 experiments sourced from 2 independent differentiations, and a total of 15 mice. Cohort #7 transplantations used PB10.5 cells from 1 differentiation experiment, and a total of 8 mice. For umbilical cord blood transplantation, 5 experiments using 4 independent cord blood samples were used to transplant a total of 39 mice. For single cell RNA seq analyses, two independent iPSC lines were profiled (RM TOM and PB1.1 BFP).
Randomization	Mice from both sexes were used in the transplantation studies. No other randomization was relevant for these studies.
Blinding	Blinding was not relevant to these studies in which the investigators correlated the differentiation conditions of the iPSCs with the engraftment outcome following transplantation.

Reporting for specific materials, systems and methods

We require information from authors about some types of materials, experimental systems and methods used in many studies. Here, indicate whether each material, system or method listed is relevant to your study. If you are not sure if a list item applies to your research, read the appropriate section before selecting a response.

Materials & experimental systems

n/a	Involved in the study
☐	☒ Antibodies
☐	☒ Eukaryotic cell lines
☒	☐ Palaeontology and archaeology
☐	☒ Animals and other organisms
☒	☐ Clinical data
☒	☐ Dual use research of concern

Methods

n/a	Involved in the study
☒	☐ ChIP-seq
☐	☒ Flow cytometry
☒	☐ MRI-based neuroimaging

Antibodies

Antibodies used

For each anit-human antibody, the following information is shown:
Antibody Supplier Fluorochrome Catalogue no. Antibody clone Dilution.
This information is also shown in Supplementary Table 24.

CD3 BD Biosciences BV421 563798 SK7 1:10
CD4 BD Pharmingen APC 555349 RPA-T4 1:30
CD8 BioLegend PE-Cy7 344712 SK1 1:50
CD13 Biolegend PE-Cy7 301712 WM15 1:100
CD19 BioLegend APC 302212 HIB19 1:30
CD33 BD Pharmingen APC 340474 P67.6 1:50
CD38 BD Pharmingen APC 555462 HIT2 1:50
BioLegend APC 303510 HIT2 1:50
CD34 BioLegend PE-Cy7 343516 581 1:100
BioLegend BV421 343609 581 1:30
CD43 BioLegend APC 343206 10G7 1:50
BD Pharmingen BV421 562916 1G10 1:25
CD44 BioLegend APC 103012 IM7 1:50
CD45 BioLegend BV421 304032 HI30 1:25
Biolegend FITC 304054 HI30 1:50
CD56 BD Pharmingen PE 555516 B159 1:50
CD73 BioLegend APC 344006 AD2 1:50
BioLegend BV421 344008 AD2 1:30
CD90 BD Pharmingen APC 559869 5E10 1:50
c-KIT BioLegend APC 313206 104D2 1:25
C-X-C chemokine receptor 4 (CXCR4) BioLegend BV421 306518 12G5 1:30
BioLegend PE-Cy7 306513 12G5 1:100
BioLegend APC 306510 12G5 1:30
EpCAM BioLegend APC 324207 9C4 1:50
Glycophorin A (GYPA, CD235) BD Pharmingen APC 551336 GA-R2 (HIR2) 1:2000
Vascular endothelial growth factor receptor 2 (VEGFR2 /KDR) BioLegend AF-647 338909 HKDR-1 1:10
BioLegend PE-Cy7 393008 A16085H 1:10
Vascular endothelial cadherin (VE-cadherin/CDH5) BD Pharmingen APC 561567 55-7H1 1:50
BioLegend APC 348508 BV9 1:50
sIgM BioLegend APC 401618 MM-30 1:50

Validation

All the antibodies used are commercially available with validation data provided on the data sheets for each antibody.

Eukaryotic cell lines

Policy information about cell lines and Sex and Gender in Research

Cell line source(s)

The RM TOM iPSC line was reprogrammed in our laboratory from ATCC purchased human foreskin fibroblasts.
The PB1.1 BFP, PB5.1 and PB10.5 iPSC lines were reprogrammed in our institute from the peripheral blood mononuclear cells of donors. Line PB5.1 is from a female donor, the remaining lines are derived from male donors.

Authentication

No further validation was performed on the ATCC-derived RM TOM line. The PB1.1 BFP, PB5.1 and PB10.5 lines were confirmed identical to the collected donor peripheral blood mononuclear cells using SNPduo analysis.

Mycoplasma contamination

Cell lines tested negative for mycoplasma contamination.

Commonly misidentified lines
(See ICLAC register)

Not appplicable.

Animals and other research organisms

Policy information about studies involving animals; ARRIVE guidelines recommended for reporting animal research, and Sex and Gender in Research

Laboratory animals	NOD,B6.Prkdcscid Il2rgtm1Wjl/SzJ KitW41/W41 (NBSGW) mice were sourced from JAX Mice and Services (stock number 0266220) at The Jackson Laboratory (Maine, USA) and a colony was established at the Murdoch Children's Research Institute.
Wild animals	The study did not involve wild animals.
Reporting on sex	Mice of both sexes were used in these experiments. The distribution of male and female recipients reflected the frequency of mice of the appropriate age available for transplantation. The sex of each transplant recipient is reported in Supplementary Tables 1, 11, 12, 14 - 17, 21.
Field-collected samples	This study did not involve samples collected from the field.
Ethics oversight	The Murdoch Children's Research Institute animal ethics committee approved all animal protocols (reference A885 and A954), and experiments were carried out under its guidelines for the care and use of laboratory animals.

Note that full information on the approval of the study protocol must also be provided in the manuscript.

Flow Cytometry

Plots

Confirm that:

☒ The axis labels state the marker and fluorochrome used (e.g. CD4-FITC).

☒ The axis scales are clearly visible. Include numbers along axes only for bottom left plot of group (a 'group' is an analysis of identical markers).

☒ All plots are contour plots with outliers or pseudocolor plots.

☒ A numerical value for number of cells or percentage (with statistics) is provided.

Methodology

Sample preparation	Suspension hematopoietic cells and disaggregated embryoid bodies from differentiatied iPSCs were analyzed by flow cytometry. Cells shed into the medium (suspension hematopoietic cells) were analyzed separately from cells dissociated from the swirling embryoid bodies. Embryoid bodies were disaggregated by 45 min incubation with 2mg/ml Collagenase Type I (Worthington) at 37C. For analysis of mouse tissues, hematopoietic cells were flushed from bone marrow, spleen and thymus using a 25G needle and 2 mL syringe with phosphate buffered saline to generate single cell suspensions. Red cell lysis of peripheral blood samples was performed by incubating 100µL of blood with 10 mL of ammonium chloride lysis buffer (155 mM NH_4Cl/ 12 mM $NaHCO_3$/ 0.1 mM EDTA) at 37C for 15 min. For analysis, all samples were resuspended in phosphate buffered saline supplemented with 2% fetal calf serum. Samples were incubated with the indicated dilution of antibodies in a volume of 25 µL of PBS/2%FCS for 15 min at 4C, washed twice with 2 mL PBS/2%FCS and resuspended in 300 µL PBS/2%FCS with 1 µg/mL propidium iodide to detect dead cells.
Instrument	Flow cytometric analysis used a four laser BD LSR Fortessa analyser (Becton Dickinson).
Software	FlowLogic 8 (Inivai Technologies) was used to analyze data and prepare figures.
Cell population abundance	The abundance of populations is indicated on the flow cytometry plots and in the relevant scatter plots with mean and standard error of the mean indicated on the figure, in the figure legend or in the text as appropriate. No populations were cell sorted in this study.
Gating strategy	As shown in Supplementary Figure 1, forward scatter height and area gates were used to exclude cell doublets and the live cells were then selected on the basis of their forward scatter (> 50 on a linear scale) and low propidium iodide staining. The use of samples lacking a stain for a particular fluorochrome were use for setting of boundaries for negative staining.

☒ Tick this box to confirm that a figure exemplifying the gating strategy is provided in the Supplementary Information.
